# Environmental and geographical factors influence the occurrence and abundance of the southern house mosquito, *Culex quinquefasciatus*, in Hawai‘i

**DOI:** 10.1038/s41598-023-49793-9

**Published:** 2024-01-05

**Authors:** Oswaldo C. Villena, Katherine M. McClure, Richard J. Camp, Dennis A. LaPointe, Carter T. Atkinson, Helen R. Sofaer, Lucas Berio Fortini

**Affiliations:** 1 Hawai’i Cooperative Studies Unit, University of Hawai’i at Hilo, Hilo, HI 96720 USA; 2 The Earth Commons Institute, Georgetown University, Washington, DC 20057 USA; 3 U.S. Geological Survey, Pacific Island Ecosystems Research Center, Hawai’i National Park, HI 96718 USA

**Keywords:** Ecology, Climate sciences, Ecology, Environmental sciences

## Abstract

Hawaiian honeycreepers, a group of endemic Hawaiian forest birds, are being threatened by avian malaria, a non-native disease that is driving honeycreepers populations to extinction. Avian malaria is caused by the parasite *Plasmodium relictum*, which is transmitted by the invasive mosquito *Culex quinquefasciatus*. Environmental and geographical factors play an important role in shaping mosquito-borne disease transmission dynamics through their influence on the distribution and abundance of mosquitoes. We assessed the effects of environmental (temperature, precipitation), geographic (site, elevation, distance to anthropogenic features), and trap type (CDC light trap, CDC gravid trap) factors on mosquito occurrence and abundance. Occurrence was analyzed using classification and regression tree models (CART) and generalized linear models (GLM); abundance (count data) was analyzed using generalized linear mixed models (GLMMs). Models predicted highest mosquito occurrence at mid-elevation sites and between July and November. Occurrence increased with temperature and precipitation up to 580 mm. For abundance, the best model was a zero-inflated negative-binomial model that indicated higher abundance of mosquitoes at mid-elevation sites and peak abundance between August and October. Estimation of occurrence and abundance as well as understanding the factors that influence them are key for mosquito control, which may reduce the risk of forest bird extinction.

## Introduction

The Hawaiian Islands are home to remarkable examples of adaptive radiation and speciation among endemic invertebrates, plants, and forest birds. These taxa are faced with continual threats from invasive species, human development, and changing climatic conditions. This is especially true for endemic Hawaiian honeycreepers (family *Fringillidae*, subfamily *Carduelinae*), a diverse group of forest birds that radiated from a small number of ancestral colonists to more than 70 morphologically and ecologically diverse species^1,2^. In the past decade, all species of Hawaiian honeycreeper have experienced declines; only 17 of over 50 historically known species of honeycreeper remain, and of these, 14 are federally listed as endangered^3,4^.

A primary cause for honeycreeper declines in Hawai‘i is their high susceptibility to avian malaria, a mosquito-borne disease caused by the introduced avian malaria parasite *Plasmodium relictum*^5–7^ and transmitted by the invasive mosquito *Culex quinquefasciatus*, the primary avian malaria vector in Hawai‘i^8–10^. *Culex quinquefasciatus* has dramatically expanded its range in the last decades and it is now established in much of the tropics and sub-tropics, including on many islands (e.g., Hawaii, the Galapagos) that once were protected from mosquitoes through their natural separation from mainland landmasses^10^. *Culex quinquefasciatus* exhibit multiple traits that promote invasiveness, including (1) adaptation and association with human environments, such as breeding in water polluted with human or animal waste in peri domestic or rural areas^11,95^, (2) diverse range of blood-meal hosts^12,97^, and 3) wide temperature optimum that enable it to thrive in a wide diversity of habitats^13^. Although *Cx. quinquefasciatus* is generally thought of as ornithophilic^96^, they also feed on diverse hosts such as humans, mammals, and reptiles^12,97^. *Culex quinquefasciatus* have a broad thermal range of transmission from 14.1 to 32.2 ℃ with an optimum temperature of 25.2℃^13^. Furthermore, *Cx. quinquefasciatus* mosquitoes need a period of 8–14 days to go from egg to adult stage when temperatures are between 24 and 28 ℃; survival rates are above 75% when temperatures are between 16 and 32 ℃, and longevity is between 50 and 90 days when temperature is between 16 and 24 ℃^14^. In addition to being a competent vector for avian malaria, *Cx. quinquefasciatus* are vectors of West Nile virus, Rift Valley fever virus, St. Louis and Japanese encephalitis viruses, and filarial worms that can cause diseases to humans and/or animals^12,98–100^.

Transmission of vector-borne diseases (VBDs) is complex and influenced by a myriad of abiotic and biotic factors^15,16^. Transmission of VBDs is directly impacted by climatic factors, particularly by temperature and precipitation and to a lesser degree by humidity and wind patterns^17,18^, and by geographic factors associated with temperature and land use such as elevation and distance to anthropogenic features, respectively^19^. Both climatic and geographic factors influence the spatial and temporal distribution, intensity, and duration of VBDs^15,16^ and avian malaria specifically^20^. Mosquito-borne diseases exhibit considerable seasonality linked to environmental factors, especially temperature and precipitation, and to the life cycles of the pathogens and the vectors that transmit them^14^. Temperature directly affects mosquito life history traits like development rate, mortality rate, biting rate, fecundity^13,21^ and parasite development rate^13,21,22^. Precipitation affects the presence, quality, and quantity of mosquito breeding habitats and thus impacts either the increase or decrease of mosquito populations^23^. Other environmental factors also influence mosquito seasonality. For example, photoperiod impacts overwintering behavior of some mosquitoes^24^. In addition, temperature plays a major role in the fitness and phenology of both vectors and parasites, leading to complex spatial and temporal patterns of distribution of vectors and VBDs^25,26^.

In Hawai‘i, temperature and rainfall, in conjunction with naturally-occurring and anthropogenic larval habitat, have been identified as key factors influencing *Cx. quinquefasciatus* populations and avian malaria transmission^27–29^. Previous work suggests that populations of *Culex* mosquitoes generally decline across an elevational gradient from mid- to high-elevation forest bird habitat, and that avian malaria transmission across a broader elevational gradient ranges from year-round in low elevation forests (< 300 m), seasonal with peaks in late summer at middle elevations (600–1200 m), with sharp declines at higher elevations (> 1500 m) where thermal constraints on the development of mosquitoes and *Plasmodium* within mosquitoes curtail malaria transmission^9,22,27,29,30^.

In this study, we explore environmental, anthropogenic, and seasonal drivers of mosquito occurrence and abundance across an altitudinal gradient on the Island of Hawai‘I using capture data of adult *Cx. quinquefasciatus*, collected across a broad landscape on the windward slopes of Mauna Loa and Kīlauea Volcanoes on the Island of Hawaii from 2002 to 2004. These data are the most extensive available on mosquito populations in natural areas in Hawai‘i^20,27,29,31^, but have posed challenges for analysis due to high numbers of non-detection events (i.e. trapping periods that yield zero captures) during sampling. Non-detection events may arise from a combination of imperfect detection and/or low mosquito densities in areas where native birds persist^32^. Reliable inferences depend on selecting an appropriate distribution for zero-inflated data^33^. In this work, we leveraged zero-inflated models which can handle large proportions of zeros while modeling non-zero data and have been increasingly used in epidemiological modeling and other fields^34,35^ to explore spatio-temporal variation in mosquito occurrence and abundance in mosquito count data with a high proportion of zeros.

To address our questions, we modeled and assessed the effects on mosquito occurrence and abundance of mean temperature, precipitation, distance to the nearest anthropogenic features (e.g., residential, farmland), and two-time lags for precipitation (one and two months prior to the sampling events). Our analysis and the resulting models are relevant to designing sampling strategies for future efforts to monitor the efficacy of proposed landscape-scale mosquito control in remote Hawaiian forests and other habitats in the world where mosquito vectors occur in low densities^36–38^. It also has value for guiding strategies for sampling vector populations with extremely low density and for controlling transmission of avian malaria in susceptible native forest birds.

## Methods

### Mosquito capture

**Figure 1:**
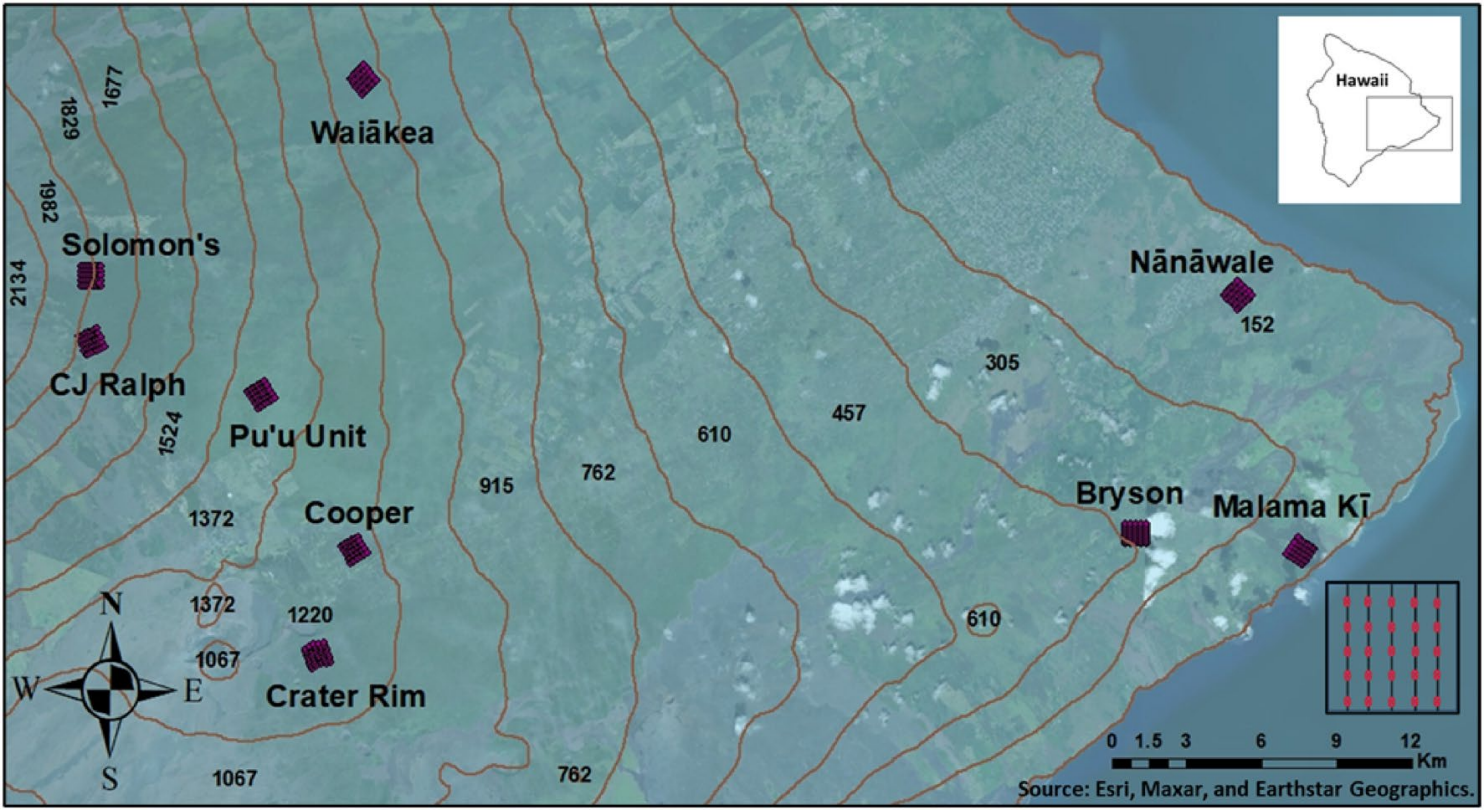
Map showing contour lines and the 9 locations where mosquito sampling took place. Low elevation sites: Bryson (BRY), Nānāwale (NAN), and Malama Kī (MAL); Middle elevation sites: Waiākea (WAI), Puʻu Unit (PUU), Cooper (COO), and Crater Rim (CRA); High elevation sites: Solomon’s (SOL) and CJ Ralph (CJR). Insets showing the study area on the Island of Hawai‘i and the mosquito sampling locations per site (transects and traps). The Map was created using ArcGIS version 10.8.2 from ESRI (https://www.esri.com/).

We collected mosquitoes at nine forested sites spanning an altitudinal gradient ranging from sea level to 1800 m on Hawai ‘i Island from February 2002 to December 2004 . Sites were located in mesic to wet forests dominated by native ‘Ōhi ‘a (*Metrosideros polymorpha*) with three low elevation (< 300 m), four middle elevation (900 – 1300 m), and two high elevation (> 1650 m) sites. At each of the nine sites, a 1 km^2^ grid was established comprising 5 transects spaced 200 m apart (Fig. 1). Each week of trapping, the traps were randomly assigned 5 out of 10 possible locations spaced at least 100 m apart along each transect. We assumed traps could attract mosquitoes within a circle with a radius of 50 m making even the closest set traps independent of each other. Mosquitoes were captured at 25 sampling locations for 3–6 trap nights every 4–6 weeks. At each mosquito sampling location, two traps were deployed: one CDC miniature light trap baited with CO_2_ (dry ice) that targets host-seeking females^39^ and one CDC gravid trap baited with an organic rich timothy-hay infusion that targets egg-laying females^40^. CDC light traps were operated without the light because in the Hawaiian rain forest, sockets corroded rapidly and lights failed regularly; in previous studies, we noticed traps without lights were catching mosquitoes similarly to those with lights. Female mosquitoes were morphologically identified by species and counted. For this study, we limit our focus to *Cx. quinquefasciatus*, the primary vector of avian malaria in Hawai ‘i. Mosquito count data were aggregated at the mosquito sampling location (i.e., trap site) and month level for the purposes of our statistical analysis (Table 1). For more details about *Cx. quinquefasciatus* trapping, identification, data storage, and to access the raw data used in this analysis, please see the U.S. Geological Survey Data Release at 10.5066/P95LVJIC^102^.

**Table 1:** Descriptive statistics of *Cx. quinquefasciatus* captures in windward Island of Hawaiʻi.

Trap type	Site	Total Count	Trap Nights	Mean	SD	% of zeroes	GIS coordinates
CO_2_	MAL	527	3330	0.61	1.78	89.7	19°26′51′′ N, 154°51′31′′ W
	NAN	1549	3252	1.74	4.38	76.9	19°32′49′′ N, 155°53′28′′ W
	BRY	768	3303	0.91	2.11	86.9	19°27′12′′ N, 154°55′18′′ W
	WAI	3777	3267	4.52	8.77	66.9	19°36′41′′ N, 155°13′30′′ W
	COO	3094	3376	3.54	5.59	65.9	19°26′36′′ N, 155°13′30′′ W
	CRA	133	3313	0.16	0.81	97.5	19°24′28′′ N, 155°14′41"W
	PUU	6268	3160	7.61	15.34	57.4	19°30′16"N,155°15′51′′ W
	CJR	22	2410	0.03	0.24	99.2	19°30′17′′ N, 155°19′28"W
	SOL	1	2743	0.00	0.04	99.9	19°32′05"N, 155°20′16′′ W
Gravid	MAL	231	3362	0.27	1.28	96.3	19°26′51′′ N, 154°51′31′′ W
	NAN	259	3333	0.29	1.32	95.9	19°32′49′′ N, 155°53′28′′ W
	BRY	351	3325	0.42	3.02	96.3	19°27′12′′ N, 154°55′18′′ W
	WAI	1063	3295	1.28	3.37	86.0	19°36′41′′ N, 155°13′30′′ W
	COO	826	3398	0.94	2.53	86.4	19°26′36′′ N, 155°13′30′′ W
	CRA	577	3347	0.69	2.58	92.5	19°24′28′′ N, 155°14′41′′ W
	PUU	170	3182	0.21	0.90	96.6	19°30′16′′ N, 155°15′51′′ W
	CJR	54	2436	0.08	0.48	98.7	19°30′17′′ N, 155°19′28′′ W
	SOL	1	2772	0.00	0.04	99.9	19°32′05′′ N, 155°20′16′′ W

Total count is the number of mosquitoes captured during the entire study period (2002–2004). Trap nights represent total nights of sampling. Mean abundance of mosquitoes and standard deviation (SD) per trap site per trapping session is shown. The percentage of zeroes represents the zeroes in the data when aggregated by trap site and trapping session as analyzed. **Low elevation sites**: Malama Kī (MAL), Nānāwale (NAN), and Bryson’s (BRY). **Middle elevation sites**: Waiākea (WAI), Pu’u Unit (PUU), Cooper’s Center (COO), and Crater Rim (CRA). **High elevation sites**: Solomon’s (SOL) and CJ Ralph (CJR).

### Environmental variables

For each mosquito sampling location, we aggregated mean monthly temperatures from a gridded 250 m resolution temperature dataset from 1990 to 2014 across the state of Hawai‘i^41^. We obtained monthly cumulative rainfall data from a gridded rainfall dataset available from 1920 to 2012 across the state of Hawai‘i^42^. We extracted monthly precipitation at each sampling location one and two months prior (i.e., the two months leading up) to each sampling period (Appendix S1: Table S1). To account for the availability of highly productive artificial larval habitats, we calculated the distance from each sampling location to the nearest anthropogenic feature (e.g., farmland, urban, roads), using a cloudless Landsat mosaic from the period between 1998 and 2002 obtained from Landsat7 satellite imagery (Appendix S1: Table S1)^43^.

### Occurrence estimation with CART and GLM

We explored drivers of occurrence of *Culex* mosquitoes across an elevational gradient using two approaches, classification and regression tree (CART) and generalized linear model (GLM)^44,45^. For each approach, we fit models with and without site as a predictor covariate (referred to here as site-specific (S) and general (G) models, respectively). Models including a covariate for site were expected to explain more variation, while models without site have more generalizability across the landscape. In both S and G models, we included predictors for trap type (CO_2_ or gravid), sampling month, monthly mean temperature (℃), monthly cumulative precipitation (mm), distance to anthropogenic features (m), and two-time lags for precipitation (1 and 2 months prior to the sampling time) to assess the effect of environmental and geographic covariates on the probability of *Cx. quinquefasciatus* occurrence. For the occurrence analysis, we treated our response variable, mosquito detection/non-detection, as a binary (0/1) response to indicate whether *Cx. quinquefasciatus* mosquitoes had been captured in the trap during that sampling period.

The CART method is a non-parametric machine learning technique applicable to both numerical and categorical data^46^. This method uses a decision tree algorithm to model the relationship between a set of input variables and a single output variable^46,106^. For this approach, we used the *rpart* function from the *rpart* package^47^ in R^48^. The CART model works by splitting the dataset recursively, which means that the subsets that result from a split (child nodes) are further split until a predetermined termination criterion is reached or until no improvement can be made. At each step, the split is based on the independent variable that results in the largest possible reduction of heterogeneity of the dependent variable to reach an impurity state of zero (i.e., the class is homogeneous) or close to zero^49,104^. To quantify the level of impurity we used the Gini index method. The Gini index reaches maximum value when all classes in the table have equal probability^49^. Child nodes terminate at leaves and each leaf in the tree diagram is labeled with the probability that the response variable (occurrence of *Culex*) is true^50^. Nodes are classified in three types: the root or parent nodes, the child nodes (derived from parent nodes), and leaf nodes which are the last nodes on a tree^51^.

We also modeled mosquito occurrence using a binomial GLM, using the *glm* function from the *stats* package in R. We fit an intercept only (null) model and a full model using the same set of predictors used in the CART method, as well as a quadratic term for temperature to accommodate a non-linear response of this variable, and an interaction term of temperature and precipitation^105,106^. We used stepwise model selection in both the forward and backwards direction to select a final model using the *step* function from the *stats* package in R. To assess GLM model assumptions, we computed and plotted the randomized quantile residuals (RQRs) in the *statmod* package^52^ in R. RQRs behave as standard normal residuals if model assumptions are met and the model adequately fits the data^53^. We compared the performance of the CART and GLM models using accuracy, precision, recall, and F1-score calculations^54,55^, as well as comparing models’ marginal prediction plots^56,57,102,103^.

### Abundance estimation with GLMM

To examine drivers of mosquito abundance, we analyzed *Cx. quinquefasciatus* count data using generalized linear mixed models, GLMMs^58,59^. GLMMs provide flexibility to analyze non-normal data and allow both fixed and random effects^58,59^. Repeated samples at the same location are often correlated, which can be accounted for using random effects^60,61^. Our fixed effects included mean temperature, squared mean temperature, precipitation, distance to anthropogenic features, trap type, two lag times for precipitation (one and two months prior to sampling), and the interaction of temperature with precipitation. We used the *glmmTMB* package^62^ in R, which accommodates a diverse set of models for zero-inflated count data and uses maximum likelihood estimation and Laplace approximation to integrate over random effects.

Our mosquito count data, particularly counts from gravid traps (Table 1), had excessive zeroes beyond what a common count distribution can accommodate, such as Poisson or negative binomial (NB). We compared zero-inflated^63,64^ and hurdle^65,66^ models, which have been developed to model zero-inflation when count models such as Poisson and NB are unfeasible^33,34,63^. The count component of a ZI model, which can include sampling zeros from non-detection, follows either a Poisson or a NB distribution^63^. When modeling the count component, a Poisson distribution can be used if the conditional mean equals the conditional variance^34,67^, in most count data sets the conditional variance is much greater than the conditional mean, a phenomenon called overdispersion, in which case a NB distribution provides a better fit^64^. In the hurdle model, the positive count data follow either truncated Poisson or truncated NB distributions^33,34,65^. We used the Akaike information criterion (AIC) for model selection^68,69^. To assess if model assumptions were adequately met, we used the *DHARMa* package^70^ to compute and plot residuals and test that a given model structure could be used to simulate the zero-inflation observed in the data.

## Results

### Mosquito trapping

During three years of sampling, a total of 19,671 *Cx.* mosquitoes were collected during 56,604 trap nights, of which 16,139 were collected using CO_2_ traps and 3,532 using gravid traps. The greatest number of mosquitoes were collected at middle elevation sites (WAI, PUU, COO, CRA) in CO_2_ (13,272 mosquitoes) or gravid (2,636 mosquitoes) traps. At low elevation sites (BRY, MAL, NAN), we captured 2,844 and 841 mosquitoes in CO_2_ and gravid traps, respectively. At high elevations, 23 mosquitoes were collected using CO_2_ traps and 55 using gravid traps (Table 1; Appendix S1: Fig. S1). Trap-level count data were strongly zero-inflated, with an average of 81% zeros in the data collected with CO_2_ traps and 94% zeros in data collected with gravid traps.

### *Culex quinquefasciatus* occurrence

#### CART model

**Figure 2:**
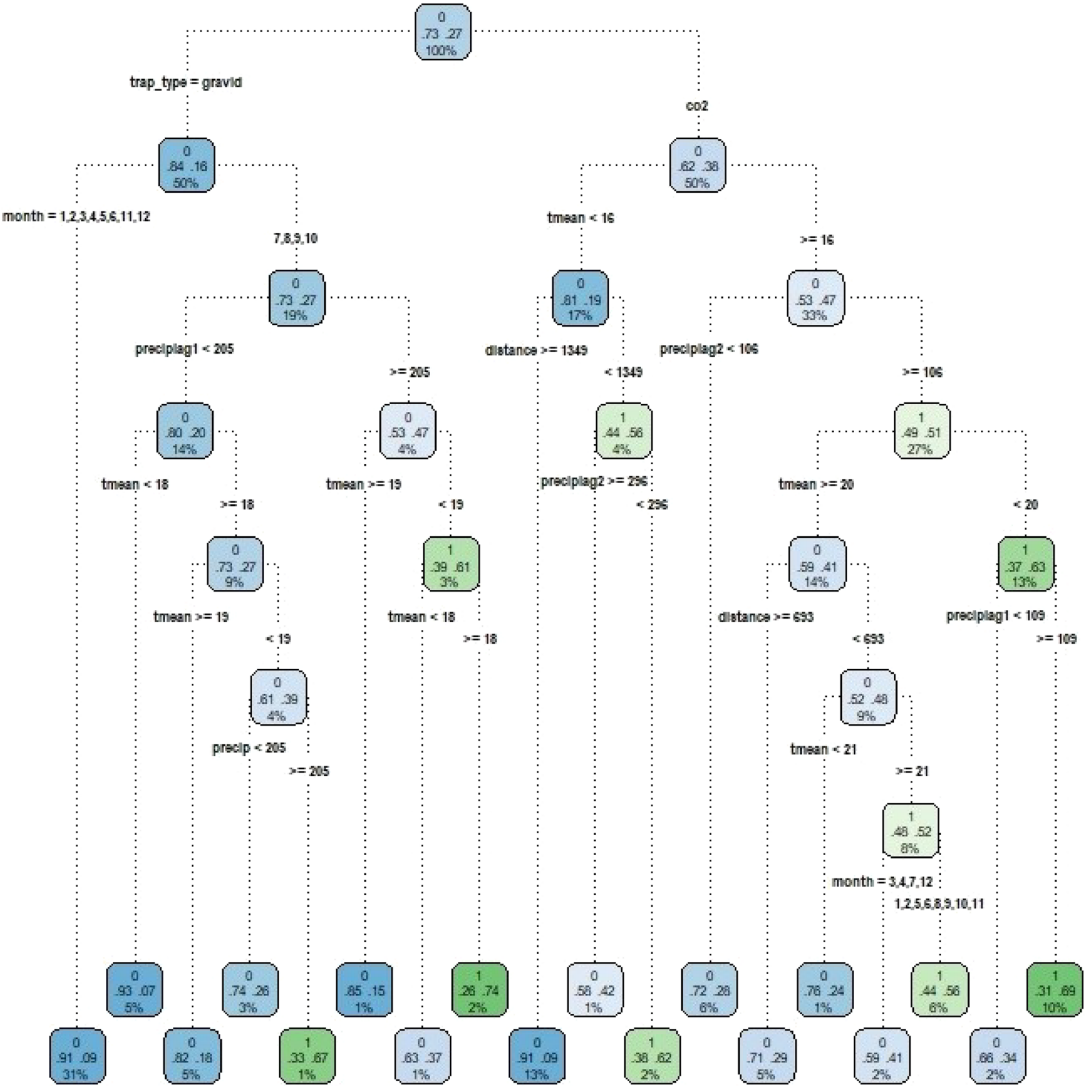
Pruned tree for the general (no site covariate) CART model showing the parameters and values that best predict *Cx. quinquefasciatus* occurrence on Hawai‘i Island. The splits from each node follow the rule left = YES. Each node contains the following information: the predicted class (detection (1) or non-detection (0)), the probability of detection/non-detection, and the percentage of observations in the node. The color scale shows the non-detection (blue) to detection (green), color changes towards white as detection/non-detection approaches 50/50 percent. tmean, mean temperature; preciplag1 and preciplag2, one and two months prior precipitation to the sampling month; distance, distance to anthropogenic features.

For model G (site not included), the initial branching depended on trap type, with lower occurrence in gravid traps compared to CO_2_ traps, and sampling month and environmental variables underlying subsequent divisions (Fig. 2). At the bottom of the tree, each leaf in the diagram shows the final probabilities of occurrence. For example, the values at the left-most leaf in Fig. 2 indicates a probability of 91% of non-detection of *Cx. quinquefasciatus* mosquitoes if the trap type is a gravid trap and the months of sampling are either January, February, March, April, May, June, November, or December. The values at the right-most leaf in Fig. 2 show a 31% probability of detection of *Cx. quinquefasciatus* mosquitoes if the trap type was a CO_2_ trap, the mean temperature was ≥ 16 ℃ and < 20 ℃, precipitation two months prior to sampling was ≥ 106 mm, and precipitation one month prior to sampling was ≥ 109 mm. For model S (including site), site was the variable that determined the initial branching, followed by trap type and sampling month (Appendix S1: section S2.1.1; Appendix S1: Fig.e S2).

### *GLM model*

**Figure 3:**
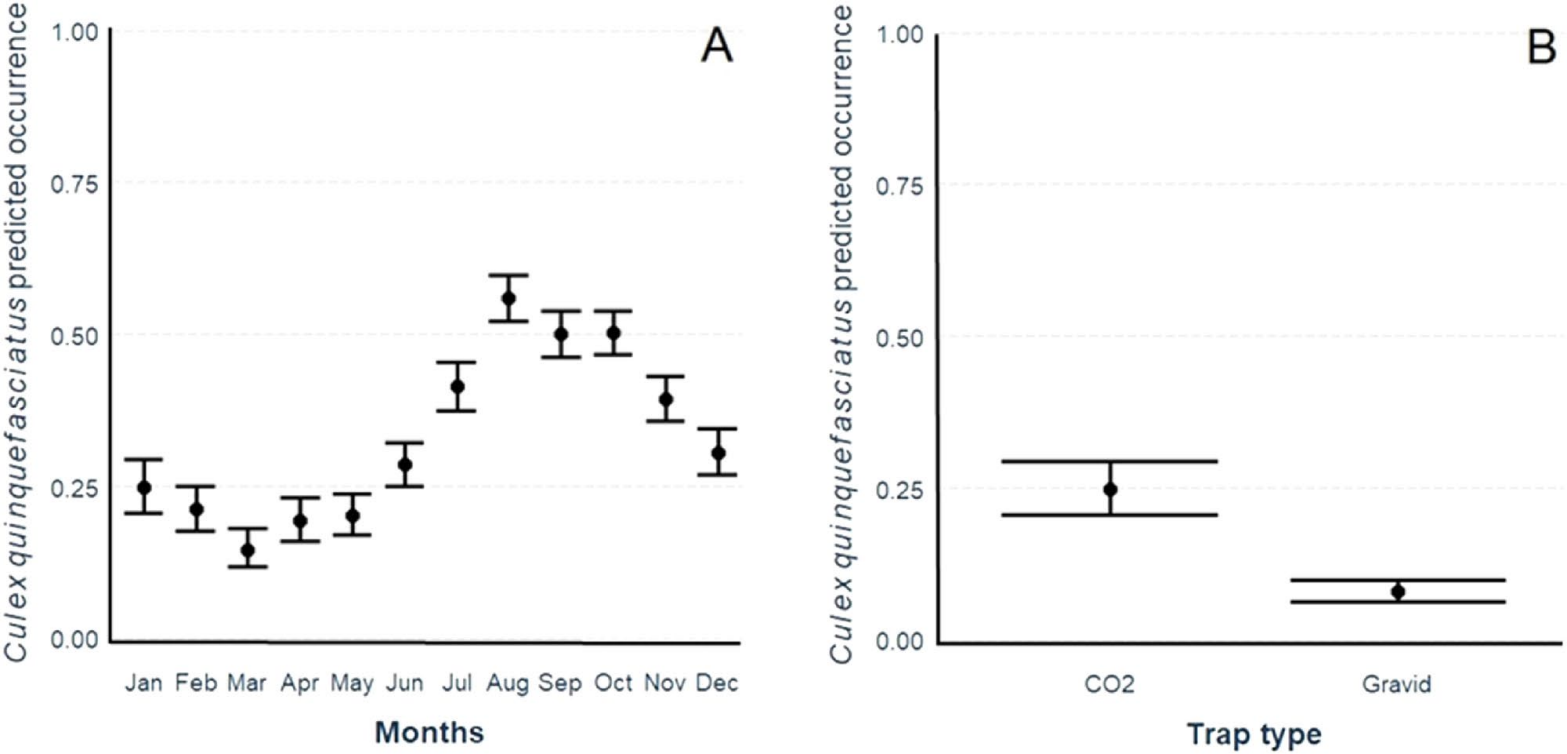
Model G: GLM model predictions for *Culex quinquefasciatus* mosquito occurrence by (**A**) month of sampling, and (**B**) trap type used during sampling.

The best general (site not included) logistic regression model to explain *Cx. quinquefasciatus* occurrence included all variables plus the quadratic temperature variable and interaction between mean temperature and monthly precipitation (Appendix S1: Table S3). Based on this final model, there was a lower probability of *Cx. quinquefasciatus* occurrence between January and May and a higher probability of *Cx. quinquefasciatus* occurrence between June to December with a peak between August and October (Fig. 3A; Appendix S1: Table S3). Gravid traps had 0.251 fewer captures of *Cx. quinquefasciatus* per trap location/month compared to CO_2_ trap location/month captures (Fig. 3B; Appendix S1: Table S3). *Culex* occurrence increased with monthly mean temperature as expected; an increase of 6.6 mosquitoes per month at each trap location for each Celsius degree increase.

Monthly precipitation and one- and two-month lags in monthly precipitation prior to the sampling month had a similar positive effect on *Culex* occurrence with an increase of 1 mosquito per one mm increase in precipitation up to 200 mm of precipitation after which mosquito occurrence levels off (Appendix S1: Table S3). Greater distance to anthropogenic features had a negative impact on *Culex* occurrence (Appendix S1: Table S3). See full description for model S (site included) in section S2.1.2 in appendix S1.

### *Comparison of occurrence model performance*

**Figure 4:**
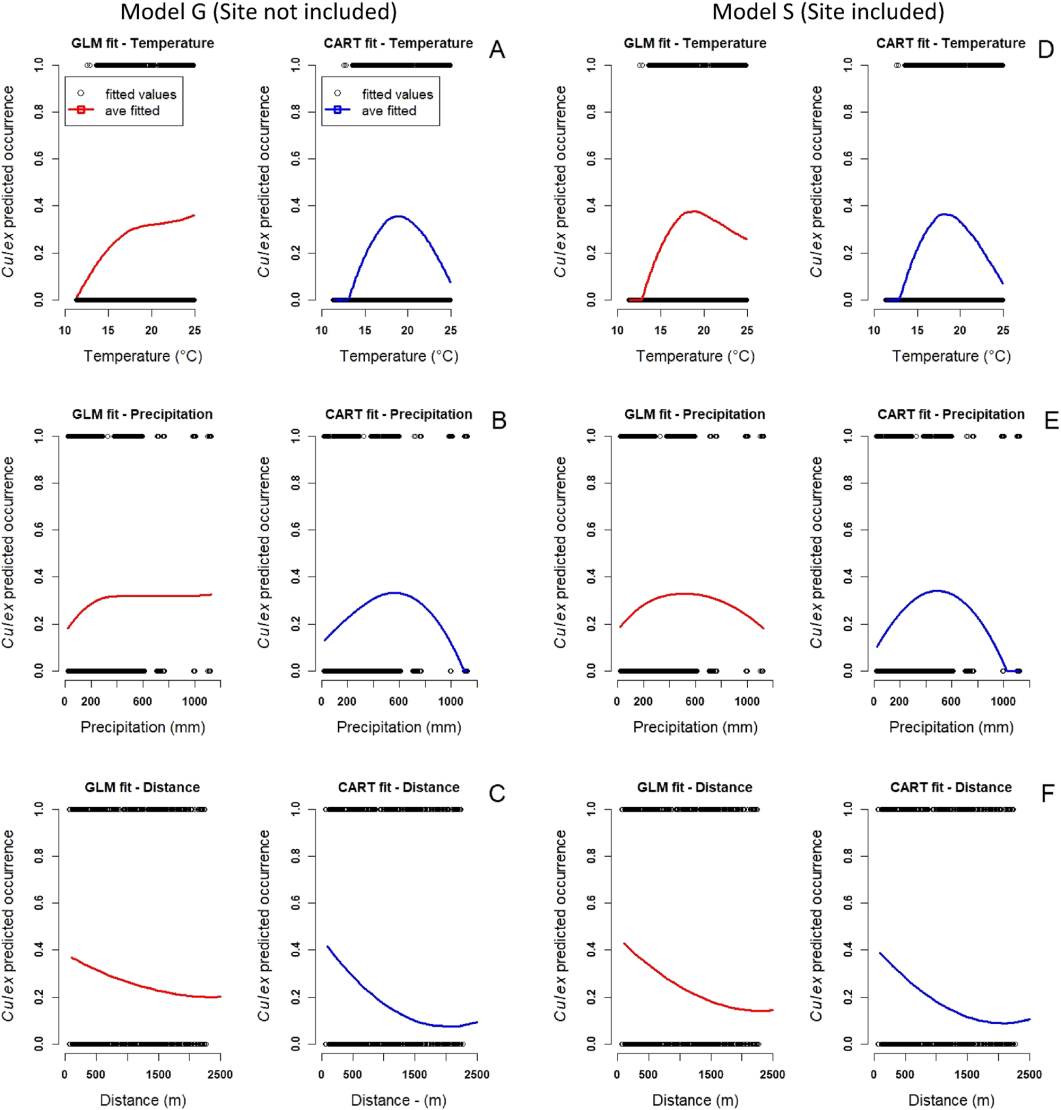
GLM and CART predictions for *Cx. quinquefasciatus* occurrence for model G (site not included) for: (**A**) temperature (°C), (**B**) precipitation, (**C**) distance to anthropogenic features and for model S (site included) for: (**D**) temperature (**°**C), (**E**) precipitation, and (**F**) distance to anthropogenic features.

Predictions from GLM models based on environmental predictors (mean temperature and precipitation) and geographical predictors (distance to anthropogenic features) did not adequately fit the non-linear nature of the count data and provided a poorer fit compared to CART models, especially for model G when the site variable was not included (Fig. 4; Appendix S1: Fig. S3). Furthermore, CART model predictions showed very little sensitivity to including site in the model, with similar responses with or without site included (Fig. 4). Tests of accuracy, precision, recall, and F1-scores validated that CART models (with and without site variable) performed better than GLM models, even after a quadratic term to account for non-linearity in temperature was included in the GLM model. The CART model G (without site) had an accuracy of 79%, a precision of 62%, a recall of 51%, and a F1-score of 56% while the GLM model G without site had an accuracy of 75%, a precision of 58%, a recall of 27%, and a F1-score of 37%. The CART model S (site included) had an accuracy of 81%, a precision of 67%, a recall of 57%, and a F1-score of 62% while the GLM model S with site included had an accuracy of 79%, a precision of 67%, a recall of 45%, and F1-score of 54% (Appendix S1: Section S4).

### *Culex quinquefasciatus* abundance

#### GLMM models

For both models G and S, a zero-inflated negative binomial distribution best described *Cx. quinquefasciatus* abundance. The zero-inflated negative binomial (ZINB) distribution was the best model based on AIC values, the diagnostic of residuals, and the ability to predict the number of zeroes in the data set (Appendix S1: Table S6, Figs. S7 and S10) when compared to the Poisson, negative binomial, hurdle models, and the zero-inflated Poisson (ZIP) distributions.

**Figure 5:**
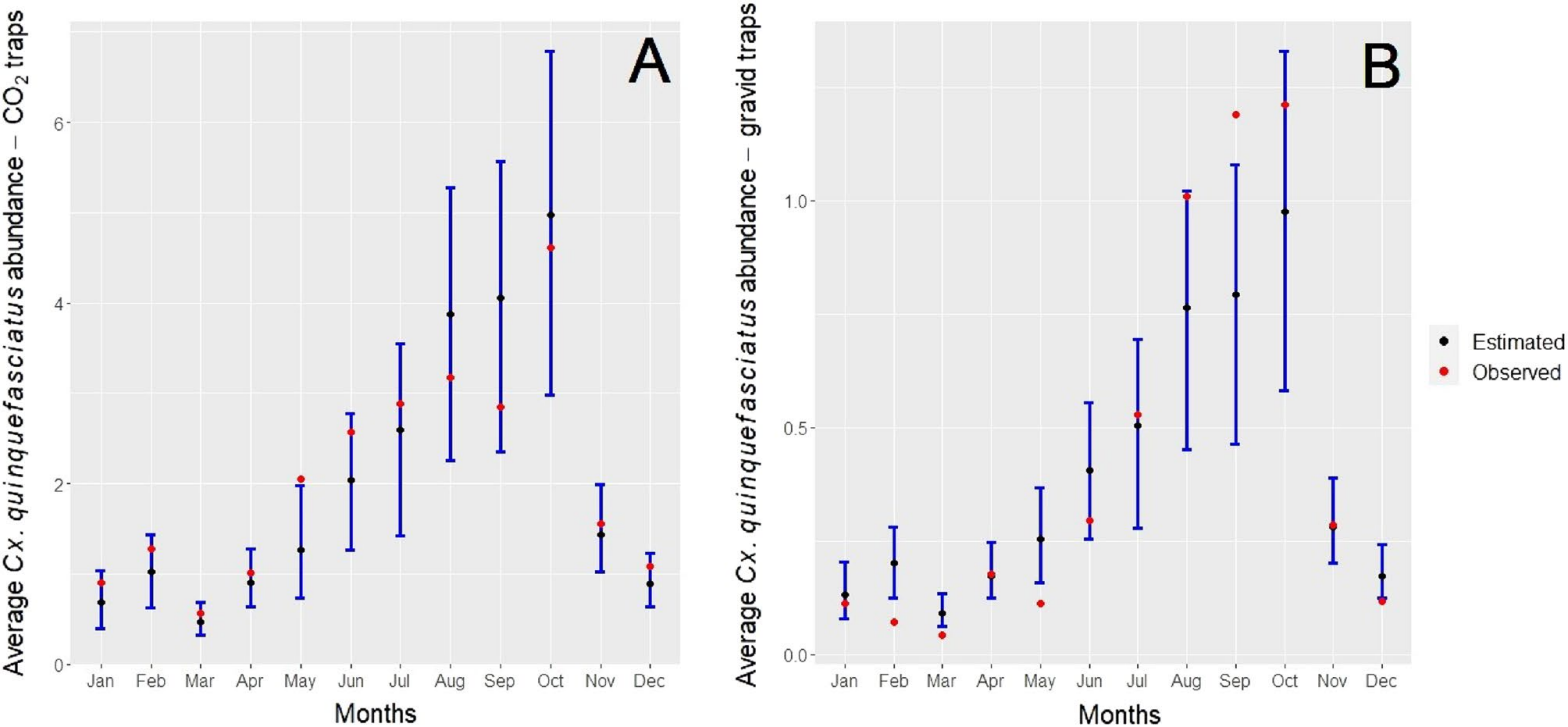
GLMM model predictions for *Cx. quinquefasciatus* mosquito abundance in Hawai‘i Island for each month using (**A**) CO_2_ traps and (**B**) gravid traps. Black dots represent predicted mean mosquito counts. Red dots represented the mean observed mosquito counts. Error bars represent the 95% confidence intervals.

For model G, the best model included month, trap type, mean temperature, the distance to anthropogenic features, precipitation in the same month, and precipitation in one and two months prior the sampling month (Appendix S1: Tables S7 and S8). The conditional part of the model (detection > 0) indicated that *Cx. quinquefasciatus* abundance showed strong seasonality (Fig. 5). Gravid traps captured fewer *Cx. quinquefasciatus* mosquitoes compared with CO_2_ traps (Appendix S1: Table S7 and Fig. S8). *Cx. quinquefasciatus* abundance increased with monthly mean temperature independent of trap type (Appendix S1: Table S7 and Fig. S8A). *Cx. quinquefasciatus* abundance decreased with monthly precipitation, especially when mosquitoes were sampled with CO_2_ traps (Appendix S1: Fig. S8B). However, precipitation one and two months prior to the sampling event had a positive effect on *Cx. quinquefasciatus* abundance (Appendix S1: Table S7). Finally, *Cx. quinquefasciatus* abundance decreased with increasing distance from anthropogenic features (Appendix S1: Fig. S8C).

For the zero-inflated portion of the model, month of sampling, monthly mean temperature, precipitation one and two months prior to the sampling event, and distance to anthropogenic features best explained non-detection of *Cx. quinquefasciatus* mosquitoes (Appendix S1: Table S8). There was low abundance of *Cx. quinquefasciatus* and frequent non-detection of mosquitoes in both traps in the months of February, March, April, and December relative to January (Fig. 5; Appendix S1: Table S8). Monthly mean temperature had a positive effect on detection of *Cx. quinquefasciatus*, with fewer detections of mosquitoes when temperatures were cooler (Appendix S1: Table S8). Also, frequency of non-detection events was higher when mosquito traps were located further from anthropogenic features (Appendix S1: Table S8).

**Figure 6:**
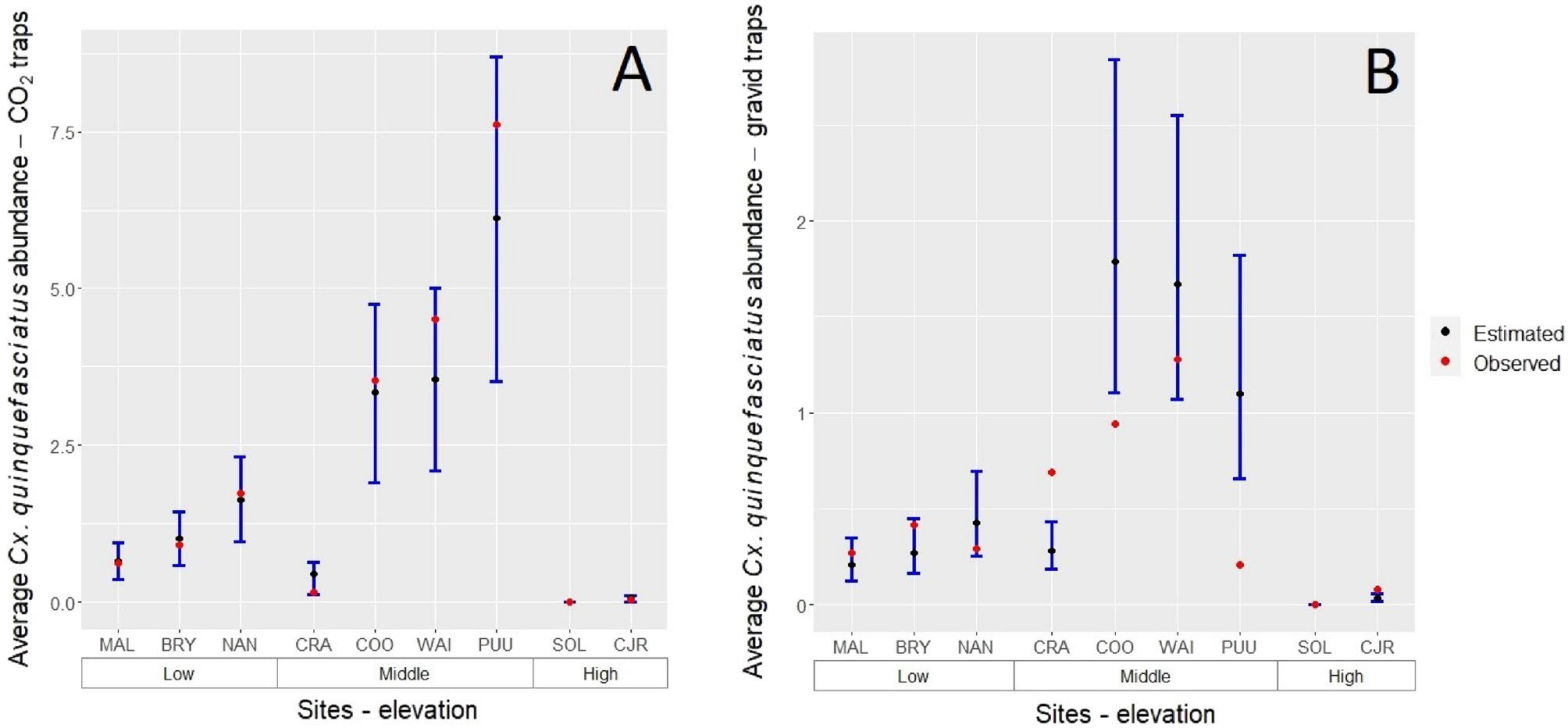
*Culex quinquefasciatus* mosquito abundance in Hawai‘i Island at three different elevation gradients (Low, Middle, and High) for each of the sampled sites using: (**A**) CO_2_ traps and (**B**) gravid traps. Black dots represent predicted mean mosquito counts. Red dots represented the mean observed mosquito counts. Error bars represent the 95% confidence intervals. Site abbreviations: BRY, Bryson; CJR, CJ Ralph; COO, Cooper; CRA, Crater Rim; MAL, Malama Kī; NAN, Nānāwale; PUU, Puʻu Unit; SOL, Solomon’s, and WAI, Waiākea.

For model S, the best model included site, month of sampling, trap type, monthly mean temperature, monthly precipitation, and distance to anthropogenic features (Appendix S1: Table S4 and S5). In the conditional part of the model (detection > 0), sites at middle elevations (PUU, WAI, and COO) had the greatest *Cx. quinquefasciatus* abundance with the exception of CRA site regardless of trap type (Fig. 6). Sites at low elevations (BRY, MAL, NAN) had on average 205% fewer *Cx. quinquefasciatus* mosquitoes than sites at middle elevations when sampled using CO_2_ traps and 300% fewer *Cx. quinquefasciatus* mosquitoes when sampled with gravid traps (Fig. 6). At high elevations, *Cx. quinquefasciatus* abundance was very low compared to low and middle elevations, especially at Solomon’s (SOL) site where only very few *Cx. quinquefasciatus* mosquitoes were captured (n = 2; Fig. 6). Trap type showed a negative effect on *Cx. quinquefasciatus* abundance with gravid traps detecting fewer mosquitoes in comparison with CO_2_ traps (Fig. 6). Similar to model G, monthly mean temperature had a positive effect on *Cx. quinquefasciatus* abundance while precipitation and distance to anthropogenic features had a negative effect on *Cx. quinquefasciatus* abundance (Appendix S1: Table S4).

## Discussion

The ability to estimate mosquito occurrence and abundance and understand their environmental and geographical drivers is critical to the success of efforts to manage mosquito populations and reduce avian malaria transmission in endemic Hawaiian forest bird populations^22^. Avian malaria has long been recognized as contributing to species extinction and population declines of endemic Hawaiian forest birds^71^. Avian malaria transmission continues to threaten native bird persistence and recovery^4,72^. Recent population declines on the islands of Kauai and Maui have been attributed to climate-change driven expansion of mosquito vectors and disease transmission at higher altitudes^11,73^. We found *Cx. quinquefasciatus* occurrence and abundance were strongly influenced by temperature and precipitation and that occurrence and abundance were highest during the warm and relatively dry months of August through October. Our results largely support earlier demographic and transmission modeling of this disease system^27,29^ and highlight the higher trap efficacy of CDC CO_2_-baited traps compared with gravid traps^74,75^. Our analytical approach was tailored to the zero-inflated nature of our dataset, providing a model for quantifying both environmental and site-specific variation in the presence and abundance of low-density mosquito populations.

We fit and evaluated multiple models of *Culex* occurrence and abundance to identify a model structure that was robust to the zero-inflation and overdispersion seen in our data and hence capable of yielding reliable and informative inferences. For occurrence, we observed a good fit between the simulation-based expected distribution of the residuals and the sample residuals from the GLM, while CART slightly outperformed the GLM based on accuracy, precision, and recall. However, both the GLM and CART models yielded similar response curves to our focal environmental variables, providing confidence in estimated relationships with mosquito occurrence using both analytical approaches. To model abundance, we evaluated zero-inflated and hurdle models, both of which provide the flexibility to model overdispersed count data with an excess of zeroes^33,76^. The best fit was achieved with a zero-inflated negative binomial model, which is a mixture of a negative binomial distribution for the counts and a point mass at zero. We note that the proportion of zeroes in our data was nevertheless associated with model fit. For example, when estimating *Cx. quinquefasciatus* abundance by site and seasonality using count data collected with CO_2_ traps (64% zeroes), we observed a smaller difference between predicted and observed values compared with results from count data collected with gravid traps (85% zeroes).

Our results showed that the occurrence and abundance of *Cx. quinquefasciatus* mosquitoes was greater at middle elevation sites, where seasonally abundant *Cx. quinquefasciatus* populations overlap with highly susceptible native bird species that function as efficient reservoir hosts for malaria^30^. Compared to middle elevation sites, low elevation sites captured two- and three-fold fewer mosquitoes on average when using CO_2_ and gravid traps, respectively. While low elevation areas are closer to thermal development optima for *Cx. quinquefasciatus*^13^ and generally closer in proximity to agricultural and residential land use that may support the highest densities of *Culex*^77,78^, our low elevation study sites were primarily located in native and non-native forest fragments where artificial and natural-occurring larval mosquito habitats were scarce^79^. Sites at high elevations had very low occurrence and abundance, especially at the highest elevation site, Solomon’s, where only two *Cx. quinquefasciatus* mosquitoes were captured during three years of sampling. Extremely low mosquito occurrence and abundance observed during this study within these high elevation forests explain in part the observed low prevalence of malaria in the remaining high elevation honeycreeper refugia^3,20^. On the eastern flank of Mauna Loa and Kilauea Volcanoes, young volcanic soils are very porous with no surface hydrology. Across this landscape, the most available larval mosquito habitat within middle elevation native forest bird habitats are naturally-occurring rainwater-filled tree fern (*Cibotium* spp.) cavities created by the destructive feeding of invasive feral pigs^11^. Other Hawaiian islands outside of our focal study area on the Island of Hawai‘i are characterized by substantially older substrates, such as those on the islands of O‘ahu and Kaua‘i. Within these landscapes, riparian habitats may be the predominant larval habitats for *Cx. quinquefasciatus,* and consequently altitudinal trends in occurrence and abundance may differ substantially from our observed patterns^73^.

Site-specific availability of habitat for larval mosquitoes likely shaped patterns of occurrence and abundance across the elevational gradient. Among our mid-elevation sites, Crater Rim had a relatively low mosquito abundance, similar to abundance observed at the low elevation sites. However, of the few mosquito captures most were in gravid traps. Crater Rim is located within Hawai‘i Volcanoes National Park where an active resource management program controls feral pigs. Larval habitat surveys revealed an absence of naturally occurring or pig-created larval mosquito habitats (personal observation, LaPointe.); probably gravid traps become more enticing in this site. Furthermore, this site is also located within a rain shadow receiving less precipitation than the other mid-elevation locations. Interestingly, the greatest mosquito abundance was observed at another middle elevation site located within Hawai‘i Volcanoes National Park (Pu’u Unit) where feral pigs were also controlled. However, water catchment and artificial containers had been placed on the site to support invasive weed control efforts and provide habitats for native damselflies and thereby inadvertently supporting high numbers of larval mosquitoes^29^. This demonstrates how the addition of even a few artificial larval habitats in native forest bird habitat can significantly influence vector abundance.

Our models showed that *Cx. quinquefasciatus* occurrence and abundance are greatly influenced by temperature. The effects of temperature were observed even after models accounted for variation among months, and response curves for occurrence estimated by CART and GLM models were strikingly similar between models (Fig. 4). The range of mean temperatures in our study (12.5–25 °C) is well within the thermal performance curve for multiple life history traits of *Cx. quinquefasciatus* mosquitoes (e.g., mosquito lifespan, fecundity, immature survival) that were calculated based on laboratory exposure of mosquitoes to different constant temperatures from 0 to 45 °C^13^. *Cx. quinquefasciatus* immature survival, lifespan, and fecundity have an optimum temperature and thermal limits of 22 °C (9–38 °C), 18 °C (NA–31 °C), and 21 °C (5–38 °C), respectively^14,80^. Multiple studies support mean temperature as a key driver of *Cx. quinquefasciatus* occurrence and abundance and the transmission of pathogens (e.g., *Plasmodium relictum*, West Nile virus) by *Cx. quinquefasciatus*^81–84^. Expected increases in temperatures due to global warming could lead to the expansion of avian malaria into high elevation forests^100,101^. Avian malaria expansion into high elevation forest could have catastrophic consequences for the few honeycreeper species that persist in high elevation thermal refugia where they have been historically protected from exposure to malaria infected mosquitoes^22^. Assessment of long-term temperature data (1917–2016) from Hawai‘i indicates that mean temperature has increased by 0.052–0.212 °C per decade since the 1910s, with 2016 showing the greatest single year increase in temperature at 0.2 °C^85–88^. At this rate of temperature increase, high elevation forests will be increasingly less safe place for native birds. In fact, recent population declines on Kauai suggest that on lower lying islands, few if any safe havens remain^73,89^. Our results support a need for continued mosquito monitoring and surveillance as climates warm.

Precipitation had weaker effects on mosquito occurrence and abundance than temperature, and we saw variability in the direction of effects across models that were dependent on the time lag. CART models predicted that *Cx. quinquefasciatus* occurrence increased with monthly precipitation, reaching a peak around 580 mm, and then decreased as precipitation increased. Estimated effects of lagged precipitation showed similar or consistently positive relationships. However, models of abundance estimated negative effects of precipitation in the month of sampling, and weak or positive effects of lagged precipitation. Some of the variation among estimates may arise from the seasonal nature of precipitation variability in Hawai‘i, where rainfall is divided into two well marked periods of six months each; a rainy and dry season^27,85^. The intensity and sequence of precipitation events can affect mosquito abundance and may contribute to the complexity in estimated relationships. For example, a heavy rainfall event may cause direct mortality to adult mosquitoes that is reflected in monthly abundance estimates. The same rainfall event may create or maintain larval mosquito habitat that will increase mosquito abundance after a lag of adequate duration to complete immature development. Intense precipitation events can also increase immature mortality by flushing larvae from their habitats^90^.

In general, both *Cx. quinquefasciatus* occurrence and abundance increased with proximity to anthropogenic features. Multiple studies have also found greater numbers of mosquitoes in anthropogenic landscapes such as urban and farmland areas^91^. Conversion of forest into urban and agriculture fields creates larval mosquito habitats, which increases mosquito occurrence and abundance^91^. In Hawai`i, the encroachment of ranchland and residential subdivisions on native forests increases *Cx. quinquefasciatus* abundance in nearby forests^28,79^.

A key result of our study is our finding that CO_2_ traps are more efficient for monitoring mosquito abundance in native Hawaiian forest bird habitats compared to gravid traps. The difference in mosquito captures between trap types (CO_2_ -baited CDC miniature versus gravid traps) is largely due to the type of baits used in each trap. CO_2_ traps attract female mosquitoes searching for a blood meal while gravid traps attract female mosquitoes in search of oviposition sites. CO_2_ traps compete with naturally occurring vertebrate hosts (e.g., birds, rodents) while gravid traps compete with natural aquatic habitats used for egg-laying. Therefore, trap efficacy may be sensitive to environmental context. For example, in low elevations the increased conversion of forested land to residential and agriculture land has created abundant artificial habitat for *Culex* mosquitoes^79^. At middle elevations, the gravid traps compete with rock pools, tree fern cavities, and ground pools created by feral pigs for oviposition sites^22,73,78^. Previous studies have shown that traps designed to attract female mosquitoes searching for a host blood meal (e.g., humans, birds, small mammals) capture greater numbers of individuals and a wider range of species compared to gravid traps^92,93^. Most studies focused on assessing distribution, occurrence, and abundance of mosquitoes use traps that simulate the host such as the CDC miniature light trap baited with CO_2_^19^. However, we note that gravid traps may be more useful for detecting malaria infection rates within mosquitoes because they acquire the parasite through feeding and have necessarily already fed when seeking an oviposition site but not when seeking a blood meal. Ultimately the type of trapping method used in mosquito monitoring programs (e.g., BG sentinel trap, CDC light trap, gravid trap, etc.) should be selected carefully to reduce the influence of competing attractants in the natural environment that could skew mosquito occurrence and abundance^93,94^.

In conclusion, our study demonstrated the effectiveness of the zero-inflated negative binomial model for representing low density mosquito capture data and the greater efficacy of CO_2_ baited traps over gravid traps. These tools will be essential for developing effective sampling strategies and to determine release rates and control efficacy of Wolbachia-based insect incompatibility control measures planned for the critical habitats of Hawai ‘i’s remaining endemic forest birds. The statistical approach we employed is also applicable to other vector monitoring and control programs. Additionally, our analysis of environmental determinants supports earlier modeling efforts on the seasonal and altitudinal occurrence and abundance of *Cx. quinquefasciatus* mosquitoes and increases our understanding of the significant role of temperature in driving mosquito abundance in Hawai‘i.

### Supplementary Information

Supplementary Information.

## Data Availability

The R code for creating model output is available publicly on Zenodo at https://zenodo.org/doi/10.5281/zenodo.10072227 and the datasets analyzed during the current study are available in the U.S. Geological Survey Data Release at 10.5066/P95LVJIC.
