# Supplementary material for: Environmental and geographical factors influence the occurrence and abundance of the southern house mosquito, Culex quinquefasciatus, in Hawai‘i
Source: Sci Rep. 2024 Jan 5;14:604. doi: 10.1038/s41598-023-49793-9 (PMC10770078; doi:10.1038/s41598-023-49793-9)
Supplement: Supplementary file 1 — Supplementary Information. [file 41598_2023_49793_MOESM1_ESM.docx]

**Supporting Information: Villena, O.C., McClure, K.M., Camp, R.J., LaPointe, D.A., Atkitson, C.T., Sofaer, H.R., and Berio Fortini, L.B. Environmental and geographical factors influence the occurrence and abundance of the southern house mosquito, *Culex quinquefasciatus*, in Hawai’i.**

**Appendix S1**

**Section S1. Mosquito counts, and descriptive statistics of geographic and climatic variables.**


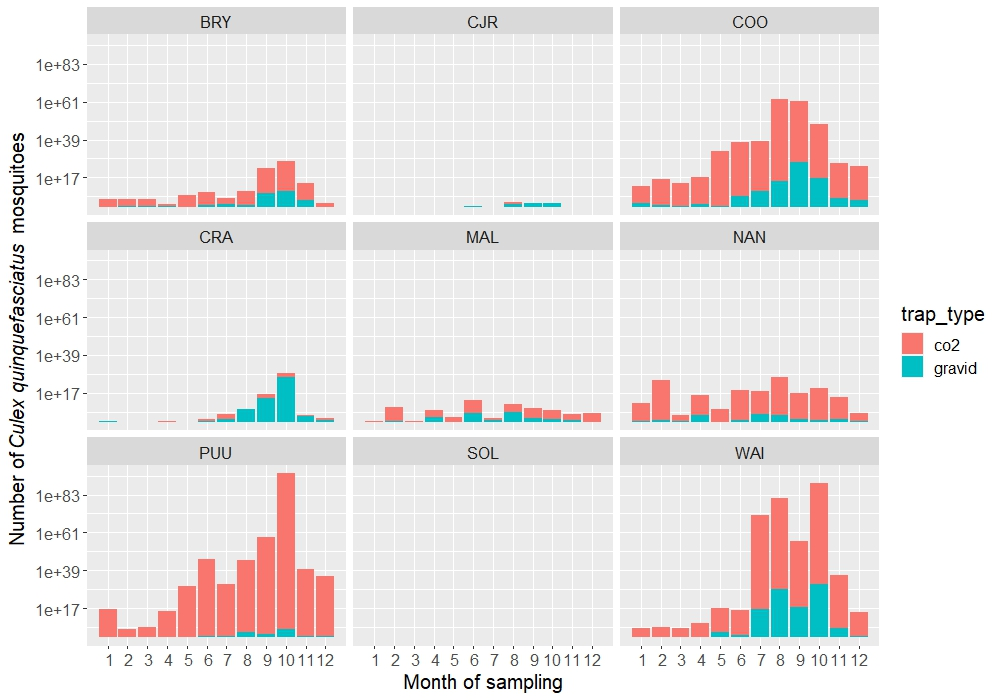


Figure S1: Observed *Culex quinquefasciatus* mosquito counts by location, month, and trap type (log scale).

Table S1: Descriptive statistics of geographic and climatic variables. Low elevation sites: Bryson (BRY), Malama-Ki (MAL), and Nanawale (NAN). Middle elevation sites: Waiakea (WAI), Pu’u Unit (PUU), Cooper (COO), and Crater Rim (CRA). High elevation sites: Solomon’s (SOL) and CJ Ralph (CJR).

| Variables | Location | Mean | Median | SD | Min. | Max. |
| --- | --- | --- | --- | --- | --- | --- |
| Elevation (m) | BRY | 290.95 | 292.15 | 12.55 | 265.39 | 314.62 |
|  | MAL | 40.77 | 41.98 | 7.15 | 28.31 | 56.26 |
|  | NAN | 65.00 | 64.62 | 5.99 | 54.71 | 75.17 |
|  | WAI | 966.29 | 965.6 | 20.63 | 929.36 | 1010.83 |
|  | PUU | 1306.87 | 1307.91 | 12.54 | 1282.86 | 1332.25 |
|  | COO | 1101.08 | 1100.79 | 13.70 | 1076.51 | 1128.82 |
|  | CRA | 1141.41 | 1140.05 | 16.14 | 1115.04 | 1175.25 |
|  | SOL | 1786.17 | 1782.25 | 25.56 | 1741.61 | 1836.10 |
|  | CJR | 1727.50 | 1726.15 | 21.70 | 1677.48 | 1775.13 |
| Distance to anthropogenic features (m) | BRY | 411.42 | 399.15 | 187.30 | 117.03 | 725.11 |
|  | MAL | 985.32 | 990.90 | 140.11 | 744.29 | 1250.17 |
|  | NAN | 493.27 | 483.06 | 154.29 | 174.26 | 793.17 |
|  | WAI | 3981.61 | 3978.55 | 286.47 | 3548.87 | 4407.57 |
|  | PUU | 1339.47 | 1341.92 | 275.26 | 776.62 | 1870.24 |
|  | COO | 325.35 | 315.22 | 138.99 | 62.46 | 606.39 |
|  | CRA | 1767.37 | 1775.75 | 271.79 | 1280.01 | 2232.75 |
|  | SOL | 1801.64 | 1781.93 | 282.14 | 1356.38 | 2268.47 |
|  | CJR | 4196.24 | 4191.18 | 268.13 | 3627.43 | 4764.17 |
| Temperature ℃ | BRY | 21.36 | 21.53 | 1.33 | 18.71 | 23.53 |
|  | MAL | 22.85 | 23.37 | 1.36 | 19.54 | 24.92 |
|  | NAN | 22.67 | 23.08 | 1.22 | 19.45 | 24.55 |
|  | WAI | 17.19 | 17.27 | 1.01 | 15.08 | 18.99 |
|  | PUU | 16.19 | 16.38 | 1.40 | 13.58 | 18.25 |
|  | COO | 16.77 | 17.00 | 1.31 | 13.57 | 18.75 |
|  | CRA | 16.98 | 17.27 | 1.30 | 13.68 | 19.01 |
|  | SOL | 13.80 | 14.02 | 1.38 | 11.31 | 16.29 |
|  | CJR | 14.21 | 14.35 | 1.39 | 11.53 | 16.70 |
| Cumulative precipitation (mm) | BRY | 211.74 | 184 | 115.65 | 56 | 610 |
|  | MAL | 171.32 | 148 | 96.22 | 49 | 534 |
|  | NAN | 207.52 | 196 | 108.45 | 44 | 607 |
|  | WAI | 412.83 | 383 | 241.49 | 50 | 1125 |
|  | PUU | 181.36 | 143 | 120.31 | 30 | 514 |
|  | COO | 224.92 | 196 | 134.04 | 29 | 594 |
|  | CRA | 156.72 | 115 | 106.99 | 21 | 540 |
|  | SOL | 148.55 | 133 | 103.60 | 22 | 441 |
|  | CJR | 142.53 | 126 | 99.45 | 25 | 458 |

**Section S2. *Culex quinquefasciatus* occurrence**

**Section S2.1. Site specific model: CART and GLM models**

**Section S.2.1.1. CART model: Pruned tree**

For the specific model (Model S), once we included the site variable in the analysis, this variable is the one that best splits this dataset. At the first level, the value of the left child node is site = BRY, CJR, CRA, MAL, SOL and the values of the right child node is site = COO, NAN, PUU, WAI. The second most important determinant for *Culex* occurrence is trap type with the left child node trap type = gravid and the right child node trap type = CO_2_. At the next level, month of sampling is the most important variable for the left (non-detection) and right (detection) child nodes with values for the left child node month = January, February, March, April, May, June, November, and December and for the child node month = January, February, March, April, and December. Next levels are described in a similar way (Figure 12).

At the end of the tree, the values at the left-most leaf in figure 18 indicates a probability of 87% of non-detection of *Cx. quinquefasciatus* mosquitoes if the site is BRY, CJR, CRA, MAl, or SOL. In the leaf before the last we observed a probability of 86% of non-detection of *Cx. quinquefasciatus* mosquitoes if the site is COO, NAN, PUU, or WAI, the trap type is a gravid trap, the month of sampling is January, February, March, April, May, June, November, or December (Figure 12). On the other hand, the values at the right-most leaf in figure 18 show a 30% probability of detection of *Cx. quinquefasciatus* mosquitoes if the site is COO, NAN, PUU, or WAI, the trap type use for sampling is a CO_2_ trap, and the months of sampling are between May and November (Figure 12). The CART model for the model S has an accuracy of 81%, a precision of 67%, a recall of 57%, and a F1-score of 62% (See section B.3.2 in the supplementary materials).


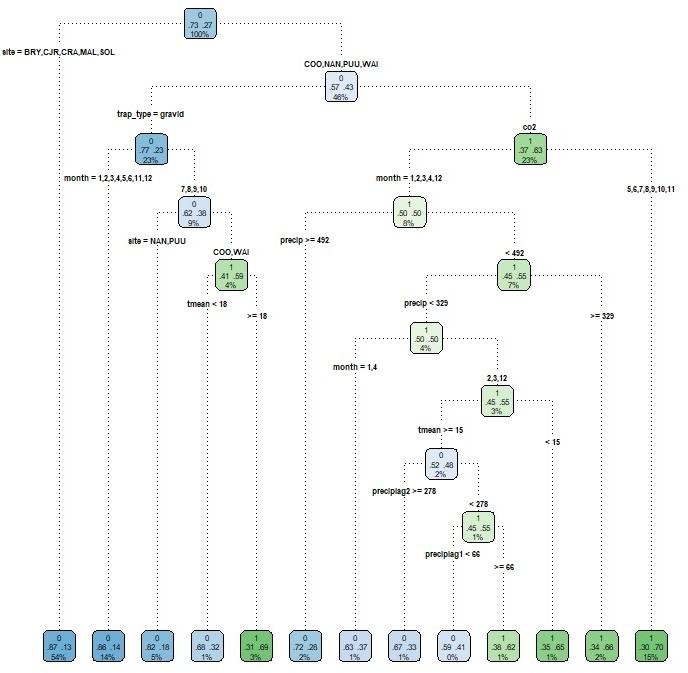


Figure S2: Pruned tree showing the parameters and values that most explain *Cx. quinquefasciatus* occurrence in Hawai‘i Island. The splits from each node follow the rule left=YES. Inside each node we have the response variable detection (1) or non-detection (0), the probability of non-detection/detection, and the percentage of the total amount of data. The color scale shows the non-detection (blue) to detection (green) state, being white when detection/non-detection is 50/50 percent.

**Section S2.1.2. GLM model: regression estimates and credible intervals**

For model S (site variable included), the best model was the one that includes the following variables: site, month of sampling, trap type, monthly mean temperature, cumulative monthly precipitation and cumulative precipitation two months prior to the sampling time (Table 4; Supplemental material). The results from the logistic model are very similar to the ones from the CART model. For example, for site, both models indicate that there is higher probability of occurrence of *Cx. quinquefasciatus* mosquitoes in COO, WAI, PUU, and NAN sites (Figures 18 and 19, Supplemental material; Figure 3A). Similarly, the results of both methods showed that there is higher probability of occurrence of *Cx. quinquefasciatus* mosquitoes between the months of July and November (Figures 18 and 19, Supplemental material). Both models showed that gravid traps capture less *Cx. quinquefasciatus* mosquitoes compared to CO_2_ traps.

Mean temperature had a positive effect on the occurrence of *Cx. quinquefasciatus* mosquitoes with an increase in occurrence of 871 mosquitoes per Celsius degree increment in temperature. Mean temperature time lag2 had a negative effect on *Cx. quinquefasciatus* mosquito’s occurrence (Table 4). Precipitation in the month of sampling has a negative effect on *Cx. quinquefasciatus* occurrence with occurrence decreasing as precipitation increases over 600 mm (Figure 13 and Table 4; Supplemental material). On the other hand, 1 and 2 months prior precipitation to the sampling month has a positive effect on *Cx. quinquefasciatus* occurrence. Finally, the interaction of mean temperature and cumulative precipitation has a positive effect on *Cx. quinquefasciatus* occurrence (Table 4; Supplemental material).

The GLM model for the model S has an accuracy of 79%, a precision of 67%, a recall of 45%, and a F1-score of 54% (See section B.2.2 in the supplementary materials).

Table S2: Site-specific model: summary of the best GLM model for *Cx. quinquefasciatus* mosquito occurrence in Hawai‘i Island.

| Variable | Estimate | S.E. |
| --- | --- | --- |
| **intercept** | **−1.046e+01** | **(1.947e+00)∗∗∗** |
| location_CJR | −6.565e−01 | (5.507e−01) |
| **location_SOL** | **−3.399e+00** | **(8.886e−01)∗∗∗** |
| **location_MAL** | **−3.634e−01** | **(1.569e−01)∗** |
| location_CRA | 2.221e−01 | (3.423e−01) |
| **location_COO** | **2.40E+00** | **(3.494e−01)∗∗∗** |
| **location_NAN** | **4.903e−01** | **(1.401e−01)∗∗∗** |
| **location_PUU** | **2.12E+00** | **(3.943e−01)∗∗∗** |
| **location_WAI** | **2.00E+00** | **(3.257e−01)∗∗∗** |
| month_February | −3.835e−02 | (1.607e−01) |
| **month_March** | **−5.394e−01** | **(1.706e−01)∗∗** |
| month_April | −2.292e−02 | (1.625e−01) |
| month_May | −2.533e−01 | (2.007e−01) |
| month_June | −1.327e−01 | (2.198e−01) |
| month_July | 1.651e−01 | (2.527e−01) |
| **month_August** | **5.974e−01** | **(2.745e−01)∗** |
| **month_September** | **6.063e−01** | **(2.485e−01)∗** |
| **month_October** | **6.056e−01** | **(2.499e−01)∗** |
| month_November | 2.756e−01 | (1.752e−01) |
| month_December | 1.148e−01 | (1.500e−01) |
| **trap type_gravid** | **−1.494e+00** | **(5.463e−02)∗∗∗** |
| **mean temperature** | **7.716e−01** | **(1.624e−01)∗∗∗** |
| **mean temperature_square** | **−1.532e−02** | **(3.864e−03)∗∗∗** |
| **cumulative precipitation** | **−2.846e−03** | **(1.438e−03)∗** |
| cumulative precipitation lag1 | 4.147e−04 | (2.197e−04). |
| **cumulative precipitation lag2** | **6.915e−04** | **(2.070e−04)∗∗∗** |
| mean temperature:cumulative precipitation | 1.181e−04 | (8.190e−05) |
| ****p <* 0*.*001; ***p <* 0*.*01; **p <* 0*.*05; *·p <* 0*.*1 | | |

**Section S2.2. General model (model G): summary GLM model**

Table S3: General model: summary of the best GLM model for *Cx.* quinquefasciatus mosquito occurrence in Hawai‘i Island.

|  | Estimate | S.E. |
| --- | --- | --- |
| **intercept** | **−1.937e+01** | **(1.142e+00)∗∗∗** |
| month_February | −2.042e−01 | (1.594e−01) |
| **month_March** | **−6.550e−01** | **(1.690e−01)∗∗∗** |
| **month_April** | **−3.157e−01** | **(1.561e−01)∗** |
| month_May | −2.581e−01 | (1.585e−01) |
| month_June | 1.947e−01 | (1.513e−01) |
| **month_July** | **7.682e−01** | **(1.453e−01)∗∗∗** |
| **month_August** | **1.35E+00** | **(1.415e−01)∗∗∗** |
| **month_September** | **1.12E+00** | **(1.405e−01)∗∗∗** |
| **month_October** | **1.12E+00** | **(1.396e−01)∗∗∗** |
| **month_November** | **6.813e−01** | **(1.400e−01)∗∗∗** |
| **month_December** | **2.922e−01** | **(1.447e−01)∗** |
| **trap type_gravid** | **−1.335e+00** | **(5.104e−02)∗∗∗** |
| **mean temperature** | **1.88E+00** | **(1.156e−01)∗∗∗** |
| **mean temperature-square** | **−4.743e−02** | **(2.873e−03)∗∗∗** |
| **cumulative precipitation** | **6.672e−03** | **(1.283e−03)∗∗∗** |
| **cumulative precipitation lag1** | **1.737e−03** | **(2.132e−04)∗∗∗** |
| **cumulative precipitation lag2** | **1.799e−03** | **(2.014e−04)∗∗∗** |
| **mean temperature: cumulative precipitation** | **−3.615e−04** | **(7.280e−05)∗∗∗** |
| **distanceto anthropogenic features** | **−2.921e−04** | **(2.433e−05)∗∗∗** |
| ∗∗∗p < 0.001; ∗∗p < 0.01; ∗p < 0.05; . p < 0.1 | | |

**Section S2.3. Comparison of CART and GLM model predictions for precipitation one and two months prior to the month of sampling (time lags)**


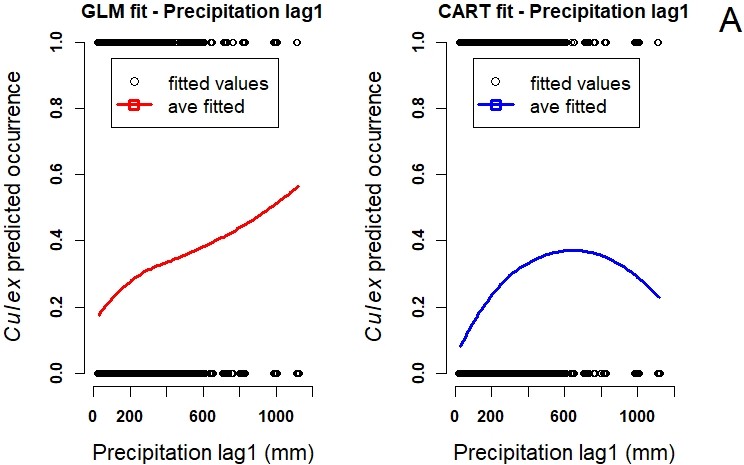

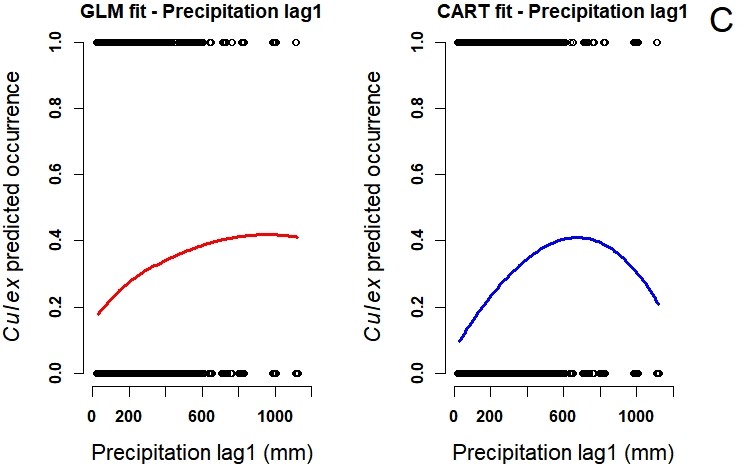


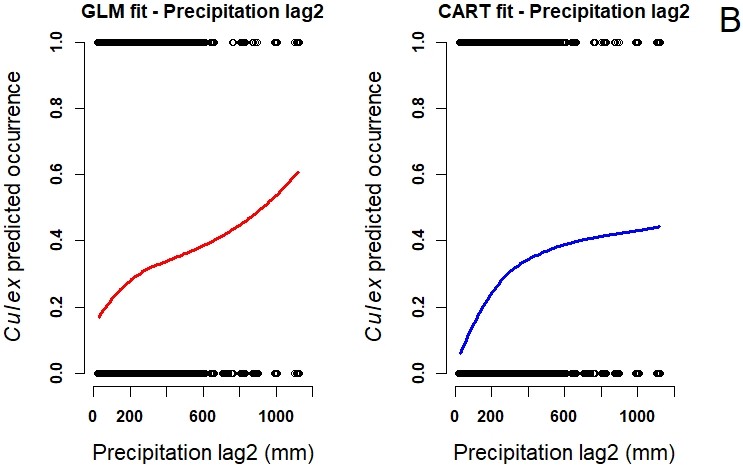

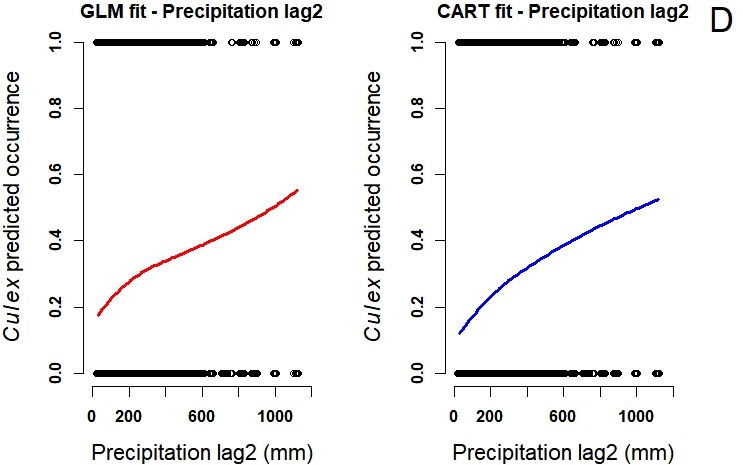


Figure S3: GLM and CART predictions for *Cx. quinquefasciatus* occurrence for model G (site no included) for: (A) precipitation lag1 (mm), (B) precipitation lag2 (mm) and for model S (site included) for: (C) precipitation lag1 (mm), (D) precipitation lag2 (mm).

**Section S2.4. Randomized quantile residuals from GLM models**


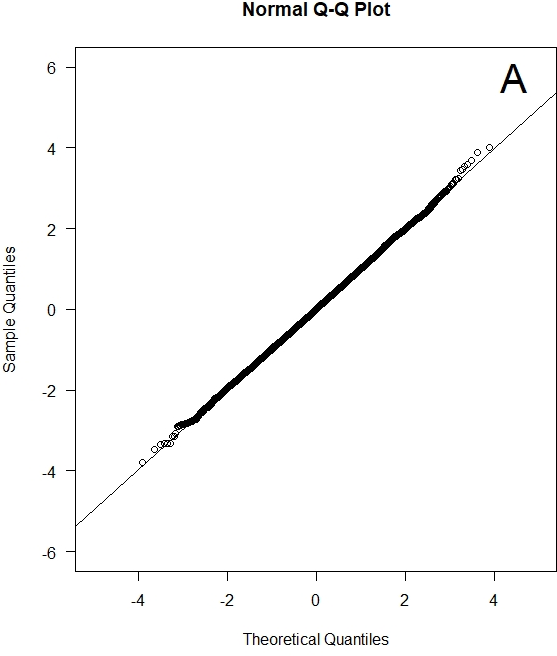

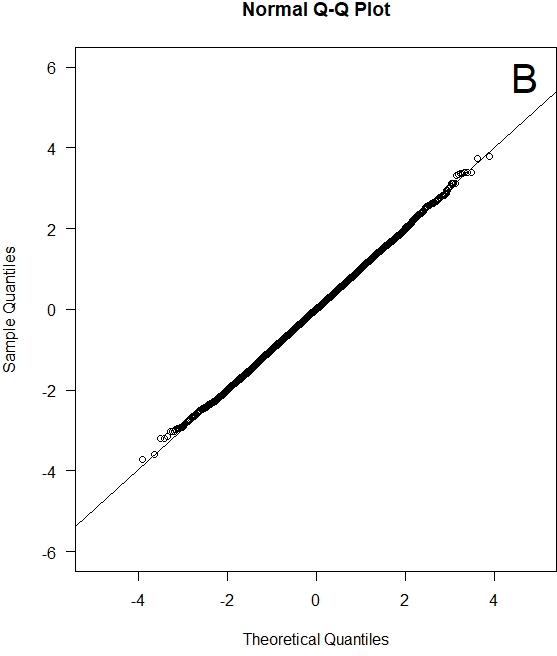


Figure S4: Q-Q plot for the quantile residuals extracted in R using *qresid* function in the package *statmod* for the best fitted model. (A) general model (B) specific model. Randomized Quantile Residuals are interpreted as standard residuals and should be normally distributed if the assumptions of the underlying model are appropriate for the data.

**Section S3. *Culex quinquefasciatus* abundance**

**Section S3.1. Specific model (model S): summary GLMM model**

Table S4: Specific model: summary of the conditional portion of the zero-inflated negative binomial model for *Cx. quinquefasciatus* mosquito abundance in Hawai‘i Island.

|  | Conditional model | |
| --- | --- | --- |
|  | Estimate | S.E. |
| **intercept** | **−9.038e+00** | **(1.581e+00)∗∗∗** |
| location_CJR | −4.946e−01 | (6.125e−01) |
| **location_COO** | **9.701e−01** | **(3.078e−01)∗∗** |
| location_CRA | −3.229e−01 | (3.391e−01) |
| location_MAL | 1.239e−01 | (1.433e−01) |
| **location_NAN** | **7.113e−01** | **(1.167e−01)∗∗∗** |
| **location_PUU** | **2.11E+00** | **(3.681e−01)∗∗∗** |
| **location_SOL** | **−5.547e+00** | **(9.106e−01)∗∗∗** |
| **location_WAI** | **3.28E+00** | **(4.428e−01)∗∗∗** |
| **month_February** | **5.289e−01** | **(1.329e−01)∗∗∗** |
| month_March | −2.274e−01 | (1.398e−01) |
| **month_April** | **3.165e−01** | **(1.338e−01)∗** |
| **month_May** | **4.352e−01** | **(1.693e−01)∗** |
| **month_June** | **6.524e−01** | **(1.822e−01)∗∗∗** |
| **Month_July** | **8.760e−01** | **(2.111e−01)∗∗∗** |
| **month_August** | **1.17E+00** | **(2.366e−01)∗∗∗** |
| **month_September** | **1.26E+00** | **(2.211e−01)∗∗∗** |
| **month_October** | **1.57E+00** | **(2.193e−01)∗∗∗** |
| **month_November** | **4.100e−01** | **(1.533e−01)∗∗** |
| month_December | 2.370e−01 | (1.260e−01). |
| **trap type_gravid** | **−3.110e−01** | **(5.937e−02)∗∗∗** |
| **mean temperature** | **9.591e−01** | **(1.232e−01)∗∗∗** |
| **mean temperature_square** | **−2.559e−02** | **(2.931e−03)∗∗∗** |
| **cumulative precipitation** | **−1.098e−03** | **(1.820e−04)∗∗∗** |
| **distance anthropogenic** | **−5.822e−04** | **(9.172e−05)∗∗∗** |
| ∗∗∗p < 0.001; ∗∗p < 0.01; ∗p < 0.05; . p < 0.1 | | |

Table S5: Specific model: summary of the zero-inflated negative binomial model for *Cx. quinquefasciatus* mosquito abundance in Hawai‘i Island.

|  | Zero-inflation model | |
| --- | --- | --- |
|  | Estimate | S.E. |
| intercept | −6.440e−01 | -1.20E+02 |
| location_CJR | 7.771e−02 | -1.86E+00 |
| **location_COO** | **−2.672e+00** | **(8.608e−01)∗∗** |
| **location_CRA** | **−2.360e+00** | **(1.012e+00)∗** |
| location_MAL | 1.752e−00 | (4.165e−01) |
| location_NAN | 1.846e−01 | (3.228e−01) |
| location_PUU | 5.497e−01 | -1.02E+00 |
| location_SOL | −3.181e+00 | -5.77E+00 |
| location_WAI | 9.842e−01 | -1.26E+00 |
| **month_February** | **9.913e−01** | **(3.910e−01)∗** |
| **month_March** | **1.27E+00** | **(4.443e−01)∗∗** |
| month_April | −2.172e−01 | (3.827e−01) |
| month_May | 6.490e−01 | (4.766e−01) |
| month_June | 7.431e−02 | (5.058e−01) |
| month_July | −5.427e−01 | (5.918e−01) |
| month_August | −9.204e−01 | (6.661e−01) |
| month_September | −9.355e−01 | (6.198e−01) |
| month_October | −9.327e−01 | (6.101e−01) |
| month_November | −5.168e−01 | (4.187e−01) |
| month_December | 2.208e−01 | (3.605e−01) |
| trap type: gravid | 1.45E+01 | -1.19E+02 |
| **mean temperature** | **−1.030e+00** | **(3.850e−01)∗∗** |
| **mean temperature_square** | **2.086e−02** | **(9.265e−03)∗** |
| cumulative precipitation | 1.675e−04 | (5.196e−04) |
| **distance anthropogenic** | **−8.534e−04** | **(2.801e−04)∗∗** |
| ∗∗∗p < 0.001; ∗∗p < 0.01; ∗p < 0.05; . p < 0.1 | | |

Table S6: A list of main effects of the main candidate models considered in the analyses of environmental and geographical effects on *Cx. quinquefasciatus* mosquitoes relative abundance, sorted by dAIC from best to least. Models were evaluated using Poisson, two Negative Binomial parameterizations, and truncated Poisson and Negative Binomial models. AIC is Akaikes’s information criterion, dAIC is the difference between AIC and the minimum AIC observed, K is the number of model parameters, and R2 conditional and R2 marginal are the conditional and marginal R2 values (Nakagawa et al., 2017). ZI: zero inflated, nbinom: negative binomial, tmean: mean temperature, precip: mean precipitation, sq: squared term, lag1 and lag 2 are one and two months previous to the mosquito collection date.

| **Family** | **Main Effects** | **Random Effects** | **AIC** | **dAIC** | **K** | **R2**  **conditional** | **R2**  **marginal** |
| --- | --- | --- | --- | --- | --- | --- | --- |
| **"zi-nbinom2"** | **site + month + trap_type + tmean + sqtmean + precip + distance*** | **(1\|year)** | **29925.73** | **0** | **52** | 0.828 | 0.815 |
| "zi-nbinom2" | site + month + trap_type + tmean + sqtmean + precip + sqprecip + distance | (1\|year) | 29946.23 | 20.50 | 54 | 0.830 | 0.817 |
| "zi-nbinom2" | site + month + trap_type + tmean + precip | (1\|year) | 30057.28 | 131.55 | 48 | 0.833 | 0.82 |
| "zi-nbinom2" | site + month + trap_type + tmean + precip + distance + preciplag1* | (1\|year) | 30057.29 | 131.56 | 52 | 0.863 | 0.856 |
| "zi-nbinom2" | site + month + trap_type* | (1\|year) | 30100.82 | 175.09 | 44 | 0.831 | 0.818 |
| "truncated-negative binomial" | site + month + trap_type + tmean + sqtmean + precip + distance* | (1\|year) | 30123.88 | 198.15 | 52 | 0.566 | 0.565 |
| "truncated-negative binomial" | site + month + trap_type + tmean + sqtmean + precip + distance + preciplag1 + preciplag2* | (1\|year) | 30133.04 | 207.31 | 56 | 0.087 | 0.075 |
| "truncated-negative binomial" | site + month + trap_type + tmean + precip + distance + preciplag1* | (1\|year) | 30172.43 | 246.7 | 52 | 0.215 | 0.214 |
| "truncated-negative binomial" | site + month + trap_type + tmean + precip + distance* | (1\|year) | 30186.93 | 261.2 | 50 | 0.566 | 0.565 |
| "truncated-negative binomial" | site + month + trap_type + tmean + precip* | (1\|year) | 30198.31 | 272.58 | 48 | 0.559 | 0.558 |
| "truncated-negative binomial" | site + month + trap_type + tmean + precip + distance + preciplag1 + preciplag2* | (1\|year) | 30205.72 | 279.99 | 54 | 0.089 | 0.088 |
| "truncated-negative binomial" | site + month + trap_type + tmean* | (1\|year) | 30211.46 | 285.73 | 46 | 0.557 | 0.557 |
| "truncated-negative binomial" | site + month + trap_type* | (1\|year) | 30242.01 | 316.28 | 44 | 0.538 | 0.537 |
| "nbinom2" | site + month + trap_type + tmean + sqtmean + precip + distance + preciplag1 + preciplag2 **^†^** | (1\|year) | 30629.61 | 703.88 | 40 | 1 | 0.986 |
| "nbinom2" | site + month + trap_type + tmean + sqtmean + precip + distance **^†^** | (1\|year) | 30634.56 | 708.83 | 38 | 1 | 0.985 |
| "nbinom2" | site + month + trap_type + tmean + sqtmean + precip + distance + preciplag1 + preciplag2 | (1\|year) | 30649.75 | 724.02 | 29 | 0.803 | 0.792 |
| "nbinom2" | site + month + trap_type + tmean + sqtmean + precip + distance | (1\|year) | 30657.92 | 732.19 | 27 | 0.803 | 0.791 |
| "nbinom2" | site + month + trap_type + tmean + precip + distance + preciplag1 **^†^** | (1\|year) | 30696 | 770.27 | 38 | 1 | 0.984 |
| "nbinom2" | site + month + trap_type + tmean + precip + distance + preciplag1 + preciplag2 **^†^** | (1\|year) | 30697.88 | 772.15 | 39 | 1 | 0.985 |
| "nbinom2" | site + month + trap_type + tmean + precip + distance **^†^** | (1\|year) | 30700.45 | 774.72 | 37 | 1 | 0.984 |
| "nbinom2" | site + month + trap_type + tmean + precip **^†^** | (1\|year) | 30728.34 | 802.61 | 36 | 1 | 0.983 |
| "nbinom2" | site + month + trap_type + tmean + precip + distance + preciplag1 | (1\|year) | 30732.6 | 806.87 | 27 | 0.79 | 0.778 |
| "nbinom2" | site + month + trap_type + tmean + precip + distance + preciplag1 + preciplag2 | (1\|year) | 30734.53 | 808.8 | 28 | 0.79 | 0.778 |
| "nbinom2" | site + month + trap_type + tmean + precip + distance | (1\|year) | 30740.36 | 814.63 | 26 | 0.79 | 0.778 |
| "nbinom2" | site + month + trap_type + tmean **^†^** | (1\|year) | 30750.31 | 824.58 | 35 | 1 | 0.985 |
| "nbinom2" | site + month + trap_type **^†^** | (1\|year) | 30754.05 | 828.32 | 34 | 1 | 0.984 |
| "nbinom2" | site + month + trap_type + tmean + precip | (1\|year) | 30773.62 | 847.89 | 25 | 0.789 | 0.776 |
| "nbinom2" | site + month + trap_type + tmean | (1\|year) | 30804.17 | 878.44 | 24 | 0.788 | 0.778 |
| "nbinom2" | site + month + trap_type | (1\|year) | 30807.56 | 881.83 | 23 | 0.788 | 0.777 |
| "zi-nbinom2" | site + month | (1\|year) | 31435.24 | 1509.51 | 42 | 0.773 | 0.76 |
| "truncated_negative binomial" | site + month | (1\|year) | 31488.57 | 1562.84 | 42 | 0.545 | 0.543 |
| "nbinom2" | site + month **^†^** | (1\|year) | 31584.81 | 1659.08 | 33 | 1 | 0.98 |
| "nbinom2" | site + month | (1\|year) | 31681.33 | 1755.6 | 22 | 0.761 | 0.748 |
| "truncated_negative binomial" | site | (1\|year) | 32223.48 | 2297.75 | 20 | 0.29 | 0.289 |
| "nbinom2" | Site **^†^** | (1\|year) | 32252.7 | 2326.97 | 22 | 1 | 0.984 |
| "nbinom2" | site | (1\|year) | 32281.29 | 2355.56 | 20 | 0.755 | 0.747 |
| "nbinom2" | site | (1\|year) | 32479.44 | 2553.71 | 11 | 0.73 | 0.721 |
| "truncated_poisson" | site + month + trap_type + tmean + precip + distance + preciplag1* | (1\|year) | 43897.74 | 13972 | 51 | 0.981 | 0.954 |
| "truncated_poisson" | site + month + trap_type + tmean + precip* | (1\|year) | 43997.03 | 14071.3 | 47 | 0.999 | 0.997 |
| "truncated_poisson" | site + month + trap_type + tmean + precip + distance* | (1\|year) | 44036.16 | 14110.4 | 49 | 0.972 | 0.918 |
| "truncated_poisson" | site + month + trap_type + tmean* | (1\|year) | 44524.73 | 14599 | 45 | 0.999 | 0.997 |
| "truncated_poisson" | site + month + trap_type* | (1\|year) | 44724.54 | 14798.8 | 43 | 0.999 | 0.997 |
| "poisson" | site + month + trap_type + tmean + sqtmean + precip + distance* | (1\|year) | 44755.06 | 14829.3 | 30 | 0.97 | 0.966 |
| "poisson" | site + month + trap_type + tmean + precip + distance* | (1\|year) | 44991.24 | 15065.5 | 29 | 0.968 | 0.964 |
| "poisson" | site + month + trap_type + tmean + precip* | (1\|year) | 45045.41 | 15119.7 | 27 | 0.968 | 0.964 |
| "poisson" | site + month + trap_type + tmean* | (1\|year) | 45423.96 | 15498.2 | 25 | 0.968 | 0.965 |
| "truncated_poisson" | site + month* | (1\|year) | 47382.92 | 17457.2 | 41 | 0.999 | 0.997 |
| "zi-poisson" | site + month + trap_type* | (1\|year) | 47677.84 | 17752.1 | 23 | 0.968 | 0.964 |
| "zi-poisson" | site + month* | (1\|year) | 49911.77 | 19986 | 21 | 0.96 | 0.956 |
| "zi-poisson" | site* | (1\|year) | 50359.76 | 20434 | 19 | 0.961 | 0.958 |
| "truncated_poisson" | site* | (1\|year) | 50389.37 | 20463.6 | 19 | 0.998 | 0.997 |
| "poisson" | site + month + trap_type + tmean + sqtmean + precip + distance + preciplag1 + preciplag2 | (1\|year) | 54811.46 | 24885.7 | 28 | 0.92 | 0.904 |
| "poisson" | site + month + trap_type + tmean + sqtmean + precip + distance | (1\|year) | 54958.22 | 25032.5 | 26 | 0.92 | 0.906 |
| "poisson" | site + month + trap_type + tmean + precip + distance + preciplag1 | (1\|year) | 55171.15 | 25245.4 | 26 | 0.916 | 0.901 |
| "poisson" | site + month + trap_type + tmean + precip + distance + preciplag1 + preciplag2 | (1\|year) | 55172.36 | 25246.6 | 27 | 0.916 | 0.901 |
| "poisson" | site + month + trap_type + tmean + precip + distance | (1\|year) | 55309.04 | 25383.3 | 25 | 0.916 | 0.902 |
| "poisson" | site + month + trap_type + tmean + precip | (1\|year) | 55348.3 | 25422.6 | 24 | 0.916 | 0.902 |
| "poisson" | site + month + trap_type + tmean | (1\|year) | 56381.07 | 26455.3 | 23 | 0.916 | 0.907 |
| "poisson" | site + month + trap_type | (1\|year) | 56537.74 | 26612 | 22 | 0.916 | 0.904 |
| "poisson" | site + month | (1\|year) | 65259.78 | 35334.1 | 21 | 0.908 | 0.895 |
| "poisson" | site | (1\|year) | 72526.65 | 42600.9 | 10 | 0.899 | 0.889 |

- Same variables were used for the zero inflated portion of the model.

**^†^** The variable month was used as a dispersion factor.

**Section S3.1.1. Specific model (model S): estimated *Cx. quinquefasciatus* mosquito abundance by site, month, and trap type**


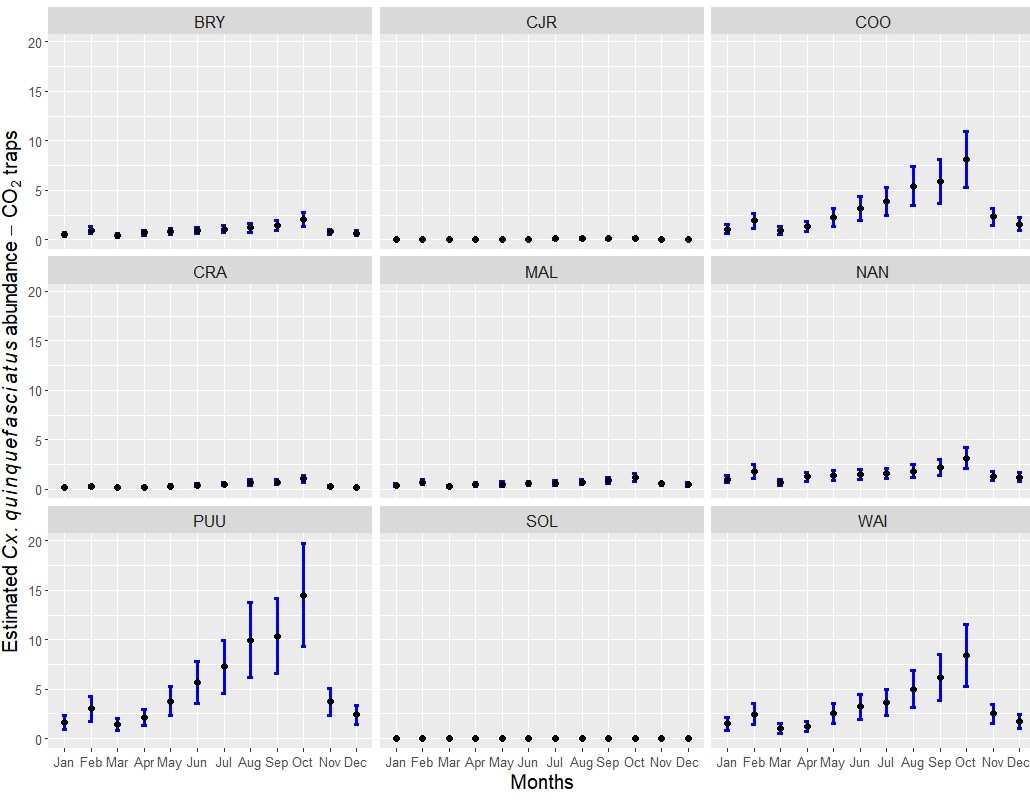


Figure S5: Estimated *Cx. quinquefasciatus* mosquito abundance in Hawai‘i Island based in the best ZINB model by month for each of the sampled sites using CO_2_ traps for sampling. Black dots represent estimated mean mosquito counts. Blue bars represent the 95% confidence intervals.


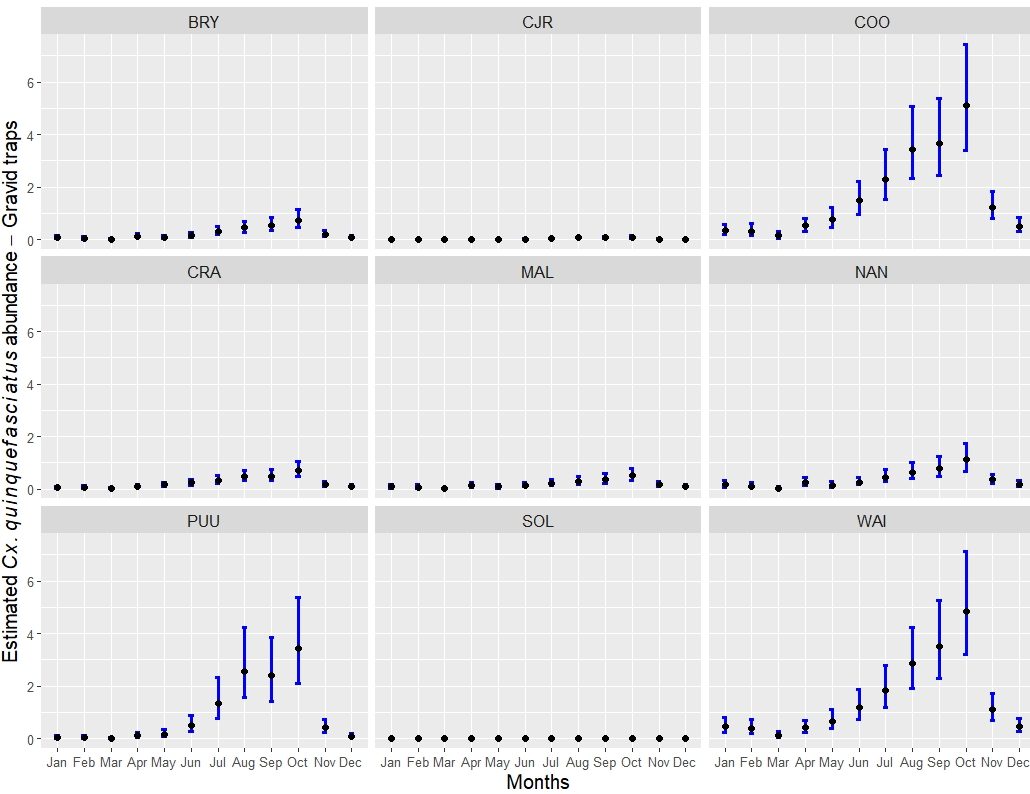


Figure S6: Estimated *Cx. quinquefasciatus* mosquito abundance in Hawai‘i Island based in the best ZINB model by month for each of the sampled sites using gravid traps for sampling. Black dots represent estimated mean mosquito counts. Blue bars represent the 95% confidence intervals.

**Section S3.1.2. Specific model (model S): diagnostic for zero-inflated negative binomial model**


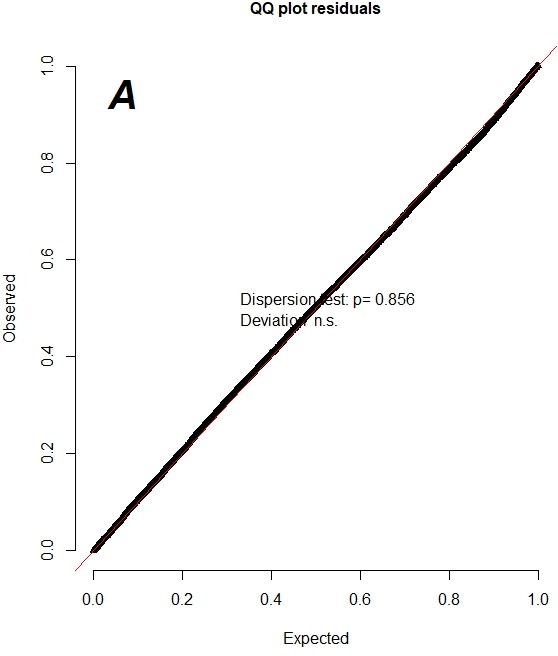

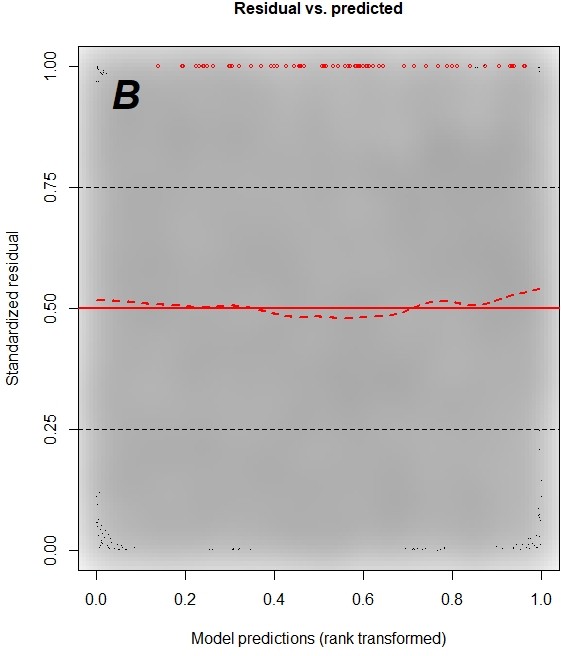

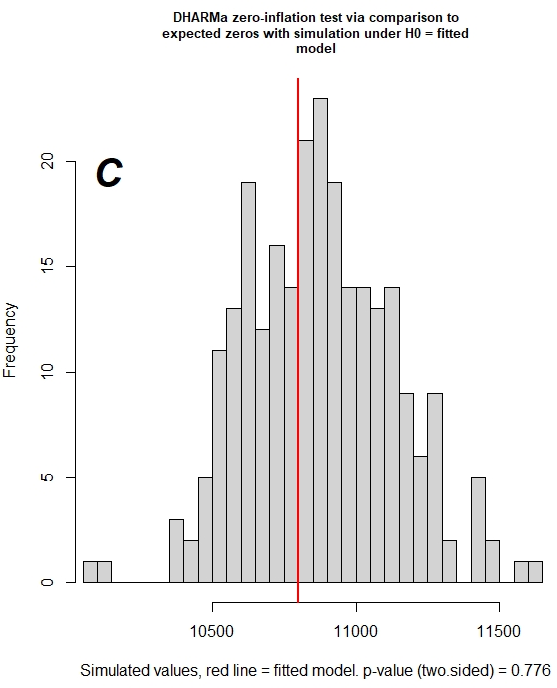


Figure S7: Specific model: diagnostic residuals and zero-inflation test. A) Q-Q plot for the quantile residuals extracted in R using the *plotQQunif* function in the package *DHARMA* for the best fitted model to detect overall deviations from the expected distribution. We also included a test for dispersion; B) Plot of the residuals against the predicted value. Outliers are highlighted as red stars; C) Check for zero-inflation where gray bars are the simulated zeroes and the red line is the amount of zeroes in the dataset.

**Section S3.2. General model (model G): summary GLMM model**

Table S7: General model: summary of the conditional portion of the zero-inflated negative binomial model for *Cx. quinquefasciatus* mosquito abundance in Hawai‘i Island.

|  | Conditional model | |
| --- | --- | --- |
|  | Estimate | S.E. |
| **intercept** | **−4.814e+00** | **(1.070e+00)∗∗∗** |
| month_February | 8.751e−02 | (1.284e−01) |
| **month_March** | **−4.280e−01** | **(1.357e−01)∗∗** |
| **month_April** | **2.605e−01** | **(1.275e−01)∗** |
| **month_May** | **3.251e−01** | **(1.290e−01)∗** |
| **month_June** | **9.764e−01** | **(1.259e−01)∗∗∗** |
| **month_July** | **1.60E+00** | **(1.218e−01)∗∗∗** |
| **month_August** | **1.95E+00** | **(1.190e−01)∗∗∗** |
| **month_September** | **1.96E+00** | **(1.198e−01)∗∗∗** |
| **month_October** | **2.22E+00** | **(1.181e−01)∗∗∗** |
| **month_November** | **8.767e−01** | **(1.194e−01)∗∗∗** |
| **month_December** | **3.440e−01** | **(1.200e−01)∗∗** |
| **trap type: gravid** | **−1.641e+00** | **(4.417e−02)∗∗∗** |
| **mean temperature** | **6.081e−01** | **(1.119e−01)∗∗∗** |
| **mean temperature_square** | **−1.950e−02** | **(2.848e−03)∗∗∗** |
| **cumulative precipitation** | **−8.843e−04** | **(1.730e−04)∗∗∗** |
| **cumulative precipitation lag1** | **1.266e−03** | **(1.870e−04)∗∗∗** |
| **cumulative precipitation lag2** | **7.838e−04** | **(1.798e−04)∗∗∗** |
| **distance anthropogenic features** | **−7.865e−05** | **(2.020e−05)∗∗∗** |
| ∗∗∗p < 0.001; ∗∗p < 0.01; ∗p < 0.05; . p < 0.1 | | |

Table S8: General model: summary of the zero-inflated negative binomial model for *Cx. quinquefasciatus* mosquito abundance in Hawai‘i Island.

|  | Zero-inflation model | |
| --- | --- | --- |
|  | Estimate | S.E. |
| **intercept** | **1.91E+02** | **(7.478e+01)∗** |
| month_February | 6.98E+00 | -6.36E+00 |
| month_March | 9.17E+00 | -6.63E+00 |
| month_April | 4.10E+00 | -5.92E+00 |
| **month_May** | **2.26E+01** | **(7.429e+00)∗∗** |
| **month_June** | **2.48E+01** | **(7.553e+00)∗∗** |
| **month_July** | **3.03E+01** | **(7.687e+00)∗∗∗** |
| **month_August** | **3.00E+01** | **(7.680e+00)∗∗∗** |
| **month_September** | **2.75E+01** | **(7.621e+00)∗∗∗** |
| **month_October** | **2.43E+01** | **(7.387e+00)∗∗** |
| **month_November** | **1.48E+01** | **(6.743e+00)∗** |
| month_December | 9.19E+00 | -6.56E+00 |
| trap type: gravid | −7.737e−01 | (4.404e−01). |
| **mean temperature** | **−2.005e+01** | **(9.840e+00)∗** |
| mean temperature_square | 4.193e−01 | (3.104e−01) |
| cumulative precipitation | −3.514e−03 | (3.987e−03) |
| **cumulative precipitation lag1** | **1.172e−02** | **(3.354e−03)∗∗∗** |
| **cumulative precipitation lag2** | **−1.758e−02** | **(3.388e−03)∗∗∗** |
| **distance anthropogenic features** | **−8.116e−04** | **(2.147e−04)∗∗∗** |
| ∗∗∗p < 0.001; ∗∗p < 0.01; ∗p < 0.05; . p < 0.1 | | |

**S3.2.1. General model: predictions of the zero-inflated negative binomial model**


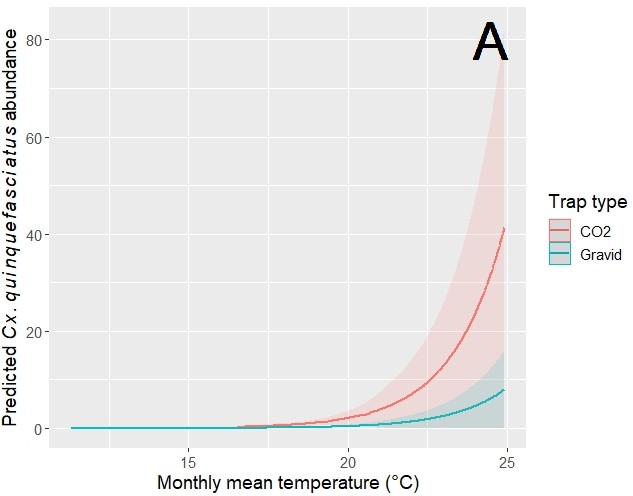

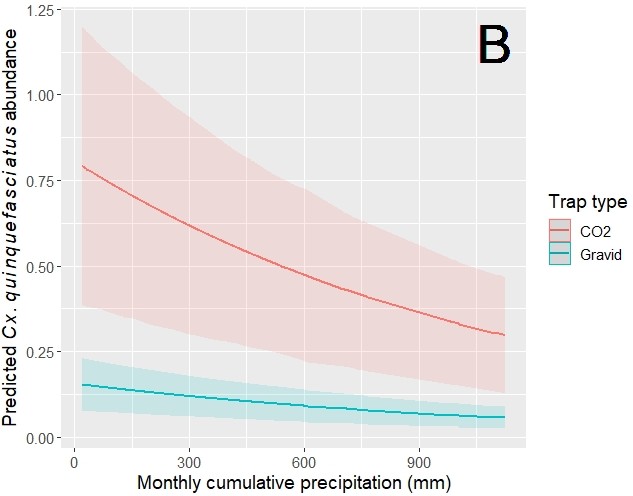


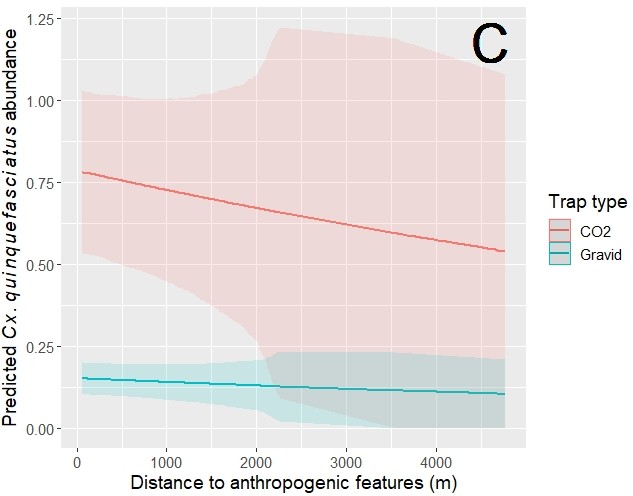


Figure S8: Model G: marginal effects of variables for best model for abundance assessment. (A) Monthly mean temperature (℃), (B) Monthly cumulative precipitation (mm), and (C) distance to anthropogenic features (m) adjusted for other variables. Average marginal effect is shown, with 95% confidence intervals. Red color is used for CO_2_ traps and blue is used for gravid traps.


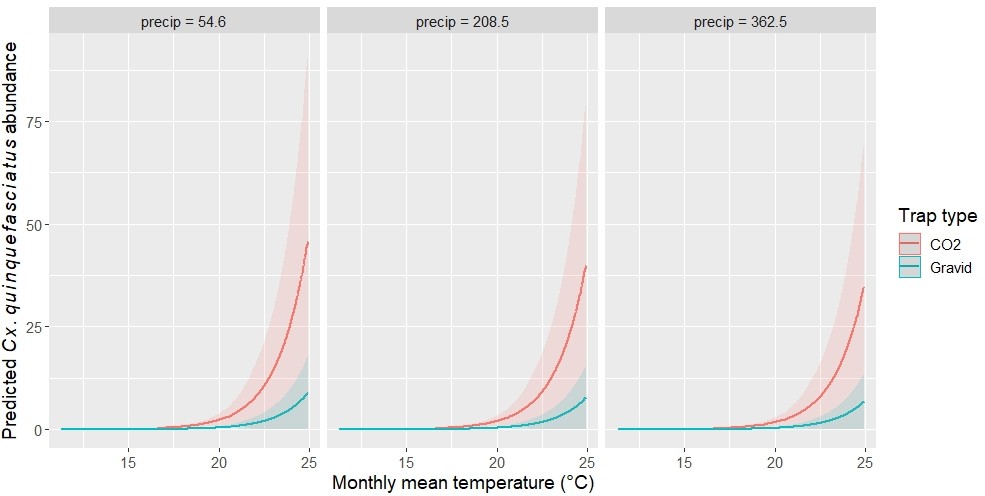


Figure S9: Model G: marginal effects for monthly mean temperature (℃) (ZINB model) at three different precipitation levels: low, medium, and high. Average marginal effect is shown, with 95% confidence intervals. Red color is used for CO_2_ traps and blue is used for gravid traps.

**S3.2.2. General model: diagnostics for zero-inflated negative binomial model**


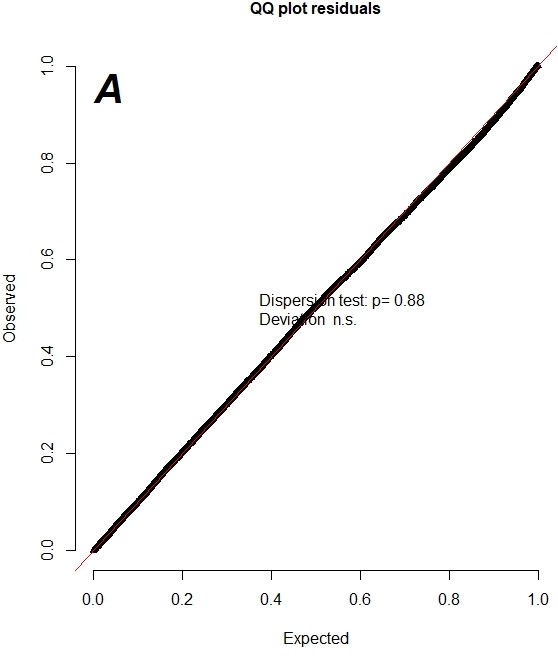

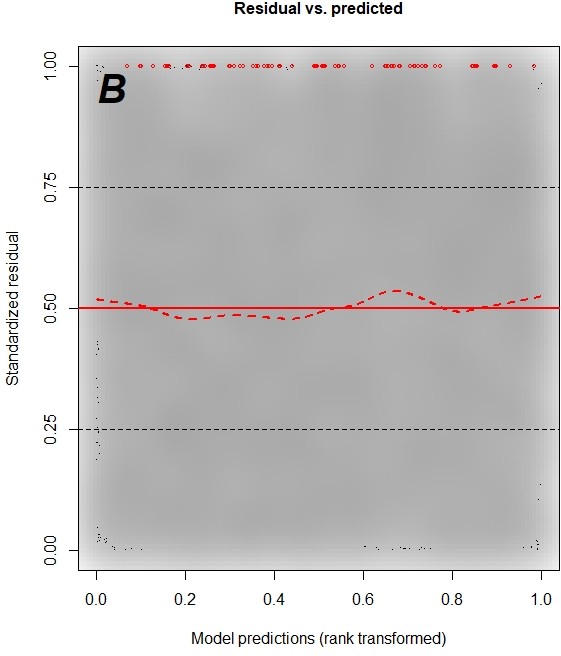

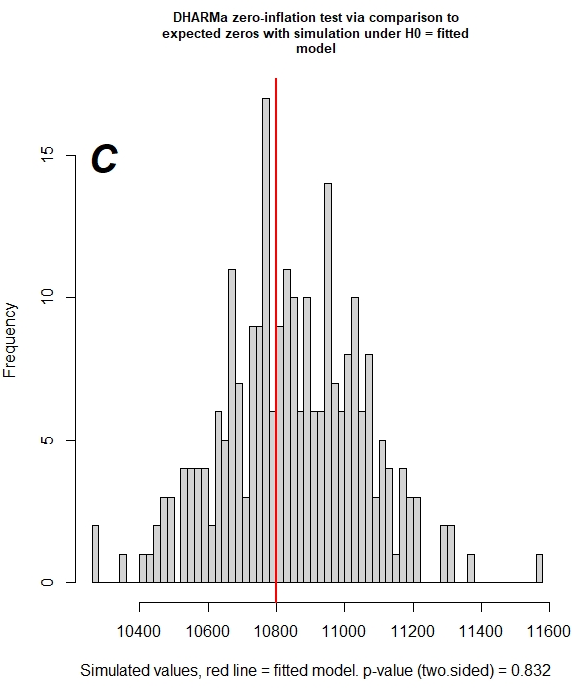


Figure S10: Residuals and zero-inflation test. A) Q-Q plot for the quantile residuals extracted in R using the *plotQQunif* function in the package *DHARMA* for the best fitted model to detect overall deviations from the expected distribution. We also included a test for dispersion; B) Plot of the residuals against the predicted value. Outliers are highlighted as red stars; C) Check for zero-inflation where gray bars are the simulated zeroes and the red line is the amount of zeros in the dataset.

**S4. Model Assessment**

**S4.1. Assessment of model performance for the specific model: CART and GLM models**

Model prediction assessment for our CART and GLM models is performed with four measurements: model accuracy, precision, recall, and the F1 score (Sofaer et al., 2019). To calculate the model accuracy, a confusion matrix is built where a count is made of the number of times “TRUE” instances are classified either as “TRUE” or “FALSE” following the next design.

Table S9: Confusion matrix.

Predicted

FALSE TRUE

Calculated FALSE True Negative (TN) False Positive (FP)

TRUE False Negative (FN) True Positive (TP)

To build the confusion matrix, a set of predictions are calculated based on the model so that they can be compared to the actual targets. To do this, we used the function *predict* in the software R, which computes the predictions on the test data set (0.3 of the total data set). In the confusion matrix, each row represents a calculated, also called actual, target, while each column represents a predicted target (Table 5). Once a confusion matrix is built, model accuracy is calculated using the following equation:

$Accuracy= \frac{TP+TN}{TP+TN+FP+FN}$ (1)

For our CART model, we built the confusion matrix shown in table 5. The first row of this matrix considers the Non-detection (0) of *Culex* mosquitoes. Of the 3122 cases, 2800 were correctly classified as non-detection of mosquitoes, while the remaining (322) was wrongly classified as non-detection of mosquitoes when mosquitoes should be detected. The second row considers the detection (1) of *Culex* mosquitoes. Of the 1145 cases, 651 were correctly classified as detection of mosquitoes, while the remaining (494) were wrongly classified as non-detection of mosquitoes when mosquitoes should be detected (Table 5). Based on these values and following equation 8 our overall model accuracy for the CART model is 81%.

Table S10: Confusion matrix for CART model. The confusion matrix is based on 4267 observations.

|  |  | Prediction | | |
| --- | --- | --- | --- | --- |
|  |  | Non-detection (0) | Detection (1) | Row total |
| Calculated | Non-detection (0) | 2800 | 322 | 3122 |
|  | Detection (1) | 494 | 651 | 1145 |
|  | Column total | 3294 | 973 | 4267 |

For our GLM model, we built the confusion matrix showed in table 6. The first row of this matrix considers the Non-detection (0) of *Culex* mosquitoes. Of the 3122 cases, 2873 were correctly classified as non-detection of mosquitoes, while the remaining (249) were wrongly classified as non-detection of mosquitoes when mosquitoes should be detected. The second row considers the detection (1) of *Cx. quinquefasciatus* mosquitoes. Of the 1145 cases, 513 were correctly classified as detection of mosquitoes, while the remaining (632) were wrongly classified as non-detection of mosquitoes when mosquitoes should be detected (Table 6). Based on these values and following equation 8, the overall model accuracy for the GLM model is 79%.

Table S11: Confusion matrix for GLM model. The confusion matrix is based on 4267 observations.

|  |  | Prediction | | |
| --- | --- | --- | --- | --- |
|  |  | Non-detection (0) | Detection (1) | Row total |
| Calculated | Non-detection (0) | 2873 | 249 | 3122 |
|  | Detection (1) | 632 | 513 | 1145 |
|  | Column total | 3505 | 762 | 4267 |

Even though our models show high accuracy, both of them overestimated the number of false negatives, especially the GLM model. This is very common when there is a dominant class which in this case is the true negatives (Non-detection) that in our data set represent the high number of zeros. In this situation, it is recommended to perform further tests such as the calculation of the model precision, the model recall, and the F1-score.

The model precision, also called positive predictive value, refers to the accuracy of the positive predictions (False and True positive). Precision is calculated using the following equation:

$Precision= \frac{TP}{TP+FP}$ (2)

The model recall, also called sensitivity, is the ratio of positive instances that are correctly detected by the classifier and it is calculated using the following equation:

$Recall= \frac{TP}{TP+FN}$ (3)

The F1-score is based on the precision and recall. The F1-score is a weighted mean of these two metrics, meaning it gives more weight to the lower values and it is calculated using the following equation:

$F1 score=2*\frac{Precision*Recall}{Precision+Recall}$ (4)

The CART model has a precision of 67%, a recall of 57%, and a F1-score of 62%, while the GLM model has a precision of 67%, a recall of 45%, and a F1-score of 54%.

**S4.2. Assessment of model performance for the general model: CART and GLM models**

The confusion matrix for our CART model showed in the first row the Non-detection (0) of *Cx. quinquefasciatus* mosquitoes. Of the 3122 cases, 2772 cases were correctly classified as non-detection of mosquitoes, while the remaining (350) were wrongly classified as non-detection of mosquitoes when mosquitoes should be detected. The second row considers the detection (1) of *Culex* mosquitoes. Of the 1145 cases, 579 were correctly classified as detection of mosquitoes, while the remaining (566) were wrongly classified as non-detection of mosquitoes when mosquitoes should be detected (Table 10). Based on these values, the CART model has an accuracy of 78.5%, a precision of 62%, a recall of 51%, and a F1-score of 56%.

Table S12: Confusion matrix for CART model without considering site variable. The confusion matrix is based on 4267 observations.

|  |  | Prediction | | |
| --- | --- | --- | --- | --- |
|  |  | Non-detection (0) | Detection (1) | Row total |
| Calculated | Non-detection (0) | 2772 | 350 | 3122 |
|  | Detection (1) | 566 | 579 | 1145 |
|  | Column total | 3338 | 929 | 4267 |

The confusion matrix for our GLM model showed in the first row the Non-detection (0) of *Cx. quinquefasciatus* mosquitoes. Of the 3122 cases, 2899 were correctly classified as non-detection of mosquitoes, while the remaining (223) were wrongly classified as detection of mosquitoes when mosquitoes should not be detected. The second row considers the detection (1) of *Culex* mosquitoes. Of the 1145 cases, 306 were correctly classified as detection of mosquitoes, while the remaining (839) were wrongly classified as non-detection of mosquitoes when mosquitoes should be detected (Table 11). Based on these values, the GLM model has an accuracy of 75%, a precision of 58%, a recall of 27%, and an F1-score of 37%.

Table S13: Confusion matrix for GLM model. The confusion matrix is based on 4267 observations.

|  |  | Prediction | | |
| --- | --- | --- | --- | --- |
|  |  | Non-detection (0) | Detection (1) | Row total |
| Calculated | Non-detection (0) | 2899 | 223 | 3122 |
|  | Detection (1) | 839 | 306 | 1145 |
|  | Column total | 3738 | 529 | 4267 |
